# On the Development of Mechanothermodynamics as a New Branch of Physics

**DOI:** 10.3390/e21121188

**Published:** 2019-12-02

**Authors:** Leonid A. Sosnovskiy, Sergei S. Sherbakov

**Affiliations:** 1 Scientific and Production Group TRIBOFATIGUE Ltd., 246050 Gomel, Belarus; tribo-fatigue@mail.ru; 2 State Committee on Science and Technology of the Republic of Belarus, 220072 Minsk, Belarus; 3 Department of Theoretical and Applied Mechanics, Belarusian State University, 220030 Minsk, Belarus

**Keywords:** mechanothermodynamics, tribo-fatigue entropy, wear-fatigue damage, stress-strain state, limiting state, damage state, dangerous volume, interaction, irreversible damage

## Abstract

This paper aims to substantiate and formulate the main principles of the physical discipline-mechanothermodynamics that unites Newtonian mechanics and thermodynamics. Its principles are based on using entropy as a bridge between mechanics and thermodynamics. Mechanothermodynamics combines two branches of physics, mechanics and thermodynamics, to take a fresh look at the evolution of complex systems. The analysis of more than 600 experimental results allowed for determining a unified mechanothermodynamical function of limiting states (critical according to damageability) of polymers and metals. They are also known as fatigue fracture entropy states.

## 1. Introduction

Any scientific discipline is based on the understanding and mathematical description of the behavior of certain phenomena revealing specific properties of some existing or imaginary objects [1,2].

**Figure 1:**
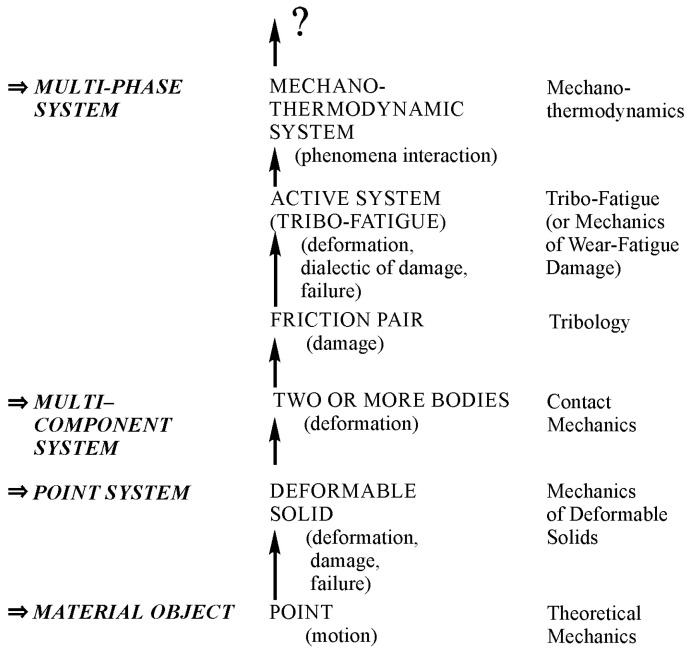
Objects of study in mechanics (from simple to complex) in a simplified hierarchical form.

Hierarchical structure of objects can be found from the study of specific objects that give rise to relevant branches of mechanics. Figure 1 shows a simplified hierarchical structure of objects (in case gas and fluid continua are absent) and mechanothermodynamics as a new branch of knowledge [2].

The concept of a material object given as a dimensionless and structureless point capable of moving in time and space gave impetus to the development of Newtonian theoretical mechanics aimed at understanding and describing a great variety of motions of such a physically unreal object. This concept made theoretical mechanics a useful science. As a result, the motion of points like electrons or planets, i.e., extremely small microcosm objects and huge universe objects can be correctly analyzed. If “big points” have mass, then the interaction patterns of moving celestial bodies, etc., in mechanics of space flight, machines, and mechanisms, all that moves, are the subject of the analysis with the implication of theoretical Newtonian mechanics methods. 

An interconnected set of points may represent a continuum—a solid, for example. When solid points are capable of moving or shifting relative to each other at different loads, it becomes possible to develop the concept of a new object, let us say a deformable solid. Naturally, mechanics of deformable solids must be developed in order to examine its stress-strain state at any point and, finally, to understand and mathematically describe changes in size, motion, and distortion of a solid as a whole. A deformable solid may be considered as a specimen, material, or a structural element in relation to the study objectives. Mechanics of materials, composites, structures, soil, etc., damage and failure mechanics (under static, cyclic, impact, loads, etc.), mesomechanics, and micromechanics, etc., examined specific properties of these objects. Mechanical behavior and properties of reversible and irreversible points motions in deformable solids were found as well by theories of elasticity and plasticity, respectively. Deformable solids also had a diversity of specific properties: viscoelasticity, elasto-viscoplasticity, etc. It was discovered that mechanics of deformable solids is one of the most powerful research means to model behavior of objects at various conditions.

One of the components of numerous mechanical systems is a deformable solid. The compression of two solids together started the development of a new branch of deformable solid mechanics–contact mechanics. Then, it is a study of a friction pair, for which a relative motion of two bodies at contact load is considered. Later, tribology as a special scientific discipline emerged. Its main objective is to examine friction behavior between solid bodies and interface damage of materials of various friction pairs at rolling, sliding, impact, slippage, etc. A friction pair may be treated as a multicomponent system since the third body forms in the region of moving contact due to the appearance of tribo-destruction products and/or the presence of lubricant. 

A “peculiar object” (active system) is more complicated than a friction pair [3]. In the twentieth century, the concept of an active system was introduced. An active system is defined by any mechanical system at cycling loading. Here, the friction process proceeds simultaneously at rolling, sliding, impact, etc. So, the active system may be considered as a friction pair, at least one element of which undergoes volumetric deformation. Such systems have complex wear-fatigue damage due to kinetic interactions of friction, fatigue, wear, corrosion, erosion, etc. Naturally, the appearance of a new object of study gave impetus to a scientific discipline shortly named tribo-fatigue (“tribo” is friction in Greek and “fatigue” is fatigue in French [3]) or mechanics of wear-fatigue damage [2] (mechanics of tribo-fatigue systems [4]).

Figure 1 displays the increase of complexity of objects that are studied by successive arrows. The last object is represented by a multi-phase system. It is a mechanothermodynamical (MTD) system uniting the laws of Newtonian mechanics and thermodynamics. The union of Newtonian mechanics and thermodynamics was formulated and experimentally proved for metals, alloys, and composites [5,6,7,8,9,10,11,12,13,14].

The above approaches and models for the energy and stress-strain states of complex systems at thermodynamic and mechanical loads are considered in the well-known works [15,16,17,18]. Damage and entropy concepts are important for building a model of an MTD system. 

The main ideas of materials’ behavior at fracture process conditions are discussed in Reference [19]. Study [20] considers features of mechanics of damage as a part of fracture mechanics and its applications. The basics of heterogeneous continuum physical mesomechanics, which develops on the border of physics of plasticity, continuum mechanics, and strength of materials, are given in Reference [21]. This discipline is concerned with stressed and damaged materials at macro-, meso- and micro-levels.

References [22,23] examine the constitutive relations for strain-induced damage at thermodynamic loads. They also discuss the use of failure mechanics of civil and mechanical engineering components in the brittle, fatigue, creep, and ductile conditions at thermomechanical loads. References [24,25] discuss the related tasks of formation plasticity and vibration theories for steady-state vibrations in elastoplastic bodies.

References [26,27] present a concise review of the main damage models for mechanics of continua and micromechanics, including evolution kinetics, and discuss further research areas. Reference [28] proposes a general development of continuum damage models. This model is defined by yield and empirical damage potential surfaces in space. It also considers damage mechanisms (cracking, isotropic damage, etc.) reducing material strength.

The stress-based limiting criterion for the conditions of linear and spatial strain states is described in Reference [29] using the results of experimental and theoretical studies. A thermodynamic model of friction and non-associated flow for geotechnical materials is given in Reference [30]. Models of large strain elastic-plastic behavior of ductile metals under anisotropic damage are investigated extensively in References [31,32]. References [33,34,35] deal with elastic, plastic, and damage behavior of materials in a thermodynamic statement using hardening internal state variables for both plasticity and damage. Some authors proposed damage theory of polycrystalline material [36,37], taking into account kinematic, thermodynamic, and kinetic coupling.

Reference [38] considers the model of microscopic damage of ellipsoidal voids that are capable of changing their shape for the materials at mixed hardening. The results of model materials X-ray tomography were used to study voids behavior in References [39,40,41]. Void growth and the shape change at large plastic deformation studied by means of scanning electron microscopy (SEM) is discussed in Reference [42]. Anisotropic damage progression for porous ductile metals with second phases is presented through mechanisms of void nucleation, growth, and coalescence in Reference [40]. Reference [43] presents the analytical and computational mesoscopic models for nucleation and interaction of microcracks near a macrocrack tip based on elasticity and dislocation theories. The framework allowing a combination of plasticity and damage models of inelastic behavior is proposed in Reference [44]. 

Generation of entropy in flow with silver and copper nanoparticles was studied in Reference [45]. Radiative mixed convective flow of viscous fluid to rotating disk was considered subject to viscous dissipation and Joule heating. It was shown that entropy generation rate increases for higher radiation parameter, Brinkman number, nanoparticle volume fraction, and Reynolds number. Entropy generation in magnetohydrodynamic radiative flow to the rotating disk of variable thickness was studied in Reference [46] and showed that entropy generation rate increases for higher radiation parameter but decreases for higher Eckert number. Another interesting study is devoted to entropy generation in nonlinear radiative flow of viscous nanomaterial towards a stretched surface [47]. An increasing trend was observed for both entropy generation and Bejan number due to the increase of thermophoresis variable and temperature difference parameter. Study of entropy generation in mixed convective flow of nanofluid between two stretchable rotating discs was made using Buongiorno nanofluid model [48] and showed that the entropy generation rate has inverse behavior in relation to the Hartman number. A study of magnetohydrodynamic radiative nanomaterial flow of Casson fluid towards a stretched surface [49] showed that entropy generation rates boost through the magnetic variable while the Bejan number decays.

References [1,50,51,52] contain the fundamentals of mechanothermodynamics and formulate two of its principles. The first principle states that damageability of all things has no conceivable boundaries. The second principle states that effective energy fluxes (entropies) at loads of different nature under irreversible changes in a MTD system are not additive, they interact dialectically. Corresponding entropy analysis [1] is made on the basic principles of tribo-fatigue [2,3,4] and thermodynamics [5]. The present study is dedicated to the analysis based on the energy presentations of mechanics, thermodynamics, and tribo-fatigue. It allowed us to reveal and study novel behavior and evolution patterns of a MTD system.

Current and perspective models and methods address the following specific features of mechanothermodynamics that differ it from thermodynamics:

1. An object (a system of interacting continuums, but not a continuum),
2. The state of the object (observed and limiting, but not just the observed),
3. Energy model (the allocation of the effective part in the irreversible component of the energy—the part spent on the production of damage, but not just the separation of energy into reversible and irreversible parts),
4. Non-additivity (the interaction of energy or entropy components caused by loads of different nature, but not their simple addition).

## 2. Thermomechanical State

We consider the thermomechanical task [15,16,17,18]. It will be used for the creation of energy and entropy models of MTD systems.

Continuum state of an elementary volume *dV* is described in the following way [16,17]:

(1) $$\sigma_{ij,j}+\rho f_{i}=\rho {\dot{v}}_{i},i=1,\;2,\;3,$$

where, the σ*_ij_* are the stresses, ρ is the density, the *f_i_* are the volumetric forces, and the *v_i_* are the velocities.

With the repeated index summation rule used, mechanical energy conservation of a continuum of volume *V* is obtained by multiplying scalar Equation (1) by a velocity vector *v_i_*:

(2) $$\int_{V}v_{i}\sigma_{ij,j}dV+\int_{V}\rho v_{i}f_{i}dV=\int_{V}\rho v_{i}{\dot{v}}_{i}dV.$$

The right side of Equation (2) is kinetic energy *K* change in the continuum of volume *V*:

(3) $$\int_{V}\rho v_{i}{\dot{v}}_{i}dV=\frac{d}{dt}\int_{V}\rho \frac{v_{i}v_{i}}{2}dV=\frac{d}{dt}\int_{V}\rho \frac{v^{2}}{2}dV=\frac{dK}{dt}.$$

Using the known transformations with the consideration of Gauss–Ostrogradsky’s theorem, we obtain the equation for continuum mechanical energy [16]:

(4) $$\frac{dK}{dt}+\int_{V}\sigma_{ij}{\dot{\varepsilon }}_{ij}dV=\int_{\Pi }\sigma_{ij}l_{j}d\Pi +\int_{V}\rho v_{i}f_{i}dV,$$

or,

$$\frac{dK}{dt}+\frac{\delta U}{dt}=\frac{\delta A}{dt},$$

where, ε*_ij_* denotes the strain rate, Π the continuum surface, *l* the director cosines at the continuum surface, δ*U*/*dt* the internal force power, and δ*A*/*dt* the power of internal surfaces and volumetric forces. 

In Expression (4), the symbol δ shows that in the general case, the increment (variation) cannot be an accurate differential. 

In the thermomechanical statement, the rate of change in the internal energy *U* [16] is usually given by the integral:

(5) $$\frac{dU}{dt}=\frac{d}{dt}\int_{V}\rho udV=\int_{V}\rho \dot{u}dV,$$

where $u=\underset{\Delta m\to 0}{\lim}\frac{u(\Delta m)}{\Delta m}$ is the specific internal energy (internal energy density) of an elementary volume of mass Δm.

The rate of heat transfer to the continuum is expressed in the following form:

(6) $$\frac{\delta Q}{dt}=-\int_{\Pi }c_{i}l_{i}d\Pi +\int_{V}\rho zdV,$$

where, *c_i_* characterizes the heat flux per unit area of the continuum surface per unit time due to heat conduction and *z–* the constant of heat radiation per unit mass per unit time.

The pattern of change in thermomechanical continuum energy is then of the form:

(7) $$\frac{dK}{dt}+\frac{dU}{dt}=\frac{\delta A}{dt}+\frac{\delta Q}{dt}$$

In Expression (7), transforming surface integrals into volume integrals yields the local form of the energy equation:

(8) $$\frac{d}{dt}\left(\frac{v^{2}}{2}+u\right)=\frac{1}{\rho }{\left(\sigma_{ij}v_{i}\right)}_{,j}+f_{i}v_{i}-\frac{1}{\rho }c_{i,i}+z.$$

If we subtract the scalar product of Equation (1) and the velocity vector *v_i_* from Equation (8), then the local energy equation will be obtained as follows:

(9) $$\frac{du}{dt}=\frac{1}{\rho }\sigma_{ij}^{}{\dot{\varepsilon }}_{ij}-\frac{1}{\rho }c_{i,i}+z=\frac{1}{\rho }\sigma_{ij}^{}{\dot{\varepsilon }}_{ij}+\frac{dq}{dt}$$

where *dq* is the heat flux per unit mass.

According to Equation (9), the internal energy changes are equal to the sum of the stress power and the heat flux to the continuum.

In relation to the thermodynamic system, we define two characteristic functions of its state: absolute temperature *T* and entropy *S* that can be interpreted as the characteristic of the ordered (or chaotic) state of the thermodynamic system. Usually, the entropy is assumed to have an additivity property, i.e.,

(10) $$S=\sum_{i}S_{i}.$$

Continuum mechanics [16,17] considers the specific entropy *S* per unit mass as:

(11) $$S=\int_{V}\rho sdV.$$

References [16,17] show that the specific entropy increment *ds* can occur because of the interaction with the environment (the increment *ds*^(*e*)^) or inside the system itself (the increment *ds*^(*i*)^):

(12) $$ds=ds^{\left(e\right)}+ds^{\left(i\right)}.$$

The quantity *ds*^(*i*)^ is equal to zero in reversible processes and is above zero in irreversible processes. 

If we express the heat flux per unit mass through *dq*, then in the case of reversible processes, the increment will be as follows:

(13) Tds=dq.

By the second law of thermodynamics, we see that the rate of change in the total entropy *S* of the continuum of volume *V* cannot be smaller than the sum of the heat flux through the volume boundary and the entropy produced by external sources inside the volume (Clausius–Duhem’s inequality) [16,17]:

(14) $$\frac{d}{dt}\int_{V}\rho sdV\geq \int_{V}\rho edV-\int_{\Pi }\frac{c_{i}l_{i}}{T}d\Pi$$

where, *e* is the local external entropy source power per unit mass. Formula (14) shows that the equality is valid for reversible processes and the inequality is valid for irreversible processes. 

In Formula (14), transforming the surface integral into the volume integral arrives at a relation for a rate of internal entropy production per unit mass:

(15) $$\gamma \equiv \frac{ds}{dt}-e-\frac{1}{\rho }{\left(\frac{c_{i}}{T}\right)}_{j}\geq 0$$

Continuum mechanics assumes that we can decompose the stress tensor into two parts: the conservative part $\sigma_{ij}^{\left(C\right)}$ for reversible processes (elastic deformation, liquid pressure) and the dissipative part $\sigma_{ij}^{\left(D\right)}$ for irreversible processes (plastic deformation, liquid viscous stresses):

(16) $$\sigma_{ij}^{}=\sigma_{ij}^{\left(C\right)}+\sigma_{ij}^{\left(D\right)}$$

We can then present an expression for energy change rate (9) in the following form:

(17) $$\frac{du}{dt}=\frac{1}{\rho }\sigma_{ij}^{}{\dot{\varepsilon }}_{ij}+\frac{dq}{dt}=\frac{1}{\rho }\sigma_{ij}^{\left(C\right)}{\dot{\varepsilon }}_{ij}+\frac{1}{\rho }\sigma_{ij}^{\left(D\right)}{\dot{\varepsilon }}_{ij}+\frac{dq}{dt}.$$

If Equation (13) is assumed to be valid for irreversible processes, then the total entropy production rate is:

(18) $$\frac{ds}{dt}=\frac{1}{\rho T}\sigma_{ij}^{\left(C\right)}{\dot{\varepsilon }}_{ij}+\frac{1}{\rho T}\sigma_{ij}^{\left(D\right)}{\dot{\varepsilon }}_{ij}+\frac{1}{T}\frac{dq}{dt},$$

or

$$\frac{ds}{dt}=\frac{1}{\rho T}\left(\frac{du_{M}}{dt}+\frac{du_{T}}{dt}\right)=\frac{1}{\rho T}\left(\frac{du_{M}^{\left(C\right)}}{dt}+\frac{du_{M}^{\left(D\right)}}{dt}+\frac{du_{T}}{dt}\right)$$

Expression (18) for the total local entropy change rate in the continuum elementary volume can find wide use in practice. 

In view of entropy additivity assumption (10), the sum in Expression (18) can be supplemented by other terms that allow the internal entropy production in the liquid (gas) volume due to different mechanisms to be taken into consideration. Similarly, for the continuum volume *dV* we can consider the internal chemical processes [15]:

(19) $$dU=dQ+dA+dU_{sub}=TdS-pdV+\sum_{1}^{n}\mu_{k}dN_{k},$$

(20) $$dS=\frac{dU+pdV}{T}-\frac{1}{T}\sum_{1}^{n}\mu_{k}dN_{k}.$$

If *dV* is considered not as a finite, but elementary volume of continuum, then based on Equations (17), (19) and (20), we can write the changes in its specific energy and entropy in the following differential form:

(21) $$du=\frac{1}{\rho }\sigma_{ij}^{}d\varepsilon_{ij}+dq+\sum_{k}^{}\mu_{k}dn_{k};$$

(22) $$ds=\frac{1}{\rho T}\sigma_{ij}^{}d\varepsilon_{ij}+\frac{1}{T}dq+\frac{1}{T}\sum_{k}^{}\mu_{k}dn_{k},$$

where, *n_k_* is the number of mols per unit mass.

For the continuum of volume *V*, on the basis of Equations (5) and (11), Expressions (21) and (22) will assume the form:

(23) $$dU=\int_{V}\rho dudV=\int_{V}\sigma_{ij}^{}d\varepsilon_{ij}dV+\int_{V}\rho dqdV+\int_{V}\rho \sum_{k}^{}\mu_{k}dn_{k}\;dV;$$

(24) $$dS=\int_{V}\rho dsdV=\frac{1}{T}\int_{V}\sigma_{ij}^{}d\varepsilon_{ij}dV+\frac{1}{T}\int_{V}\rho dqdV+\frac{1}{T}\int_{V}\rho \sum_{k}^{}\mu_{k}dn_{k}\;dV.$$

Having introduced the chemical entropy component (the last terms in Expressions (21)–(23)), we can have not only a more complete behavior of the continuum state, but we can also describe self-organization processes initiating stable structures with increasing the heat flux to the continuum.

Being quite common, the specified models of continuum energy and entropy states (Equations (17)–(24)) nevertheless do not permit one to satisfactorily describe some processes to occur in such a continuum as a deformable solid. However, a convenient idea of the additivity of energy and entropy components (Equation (11)) applicable to model elastic deformation is not suitable to describe non-linear processes. The available models do not also take into account an entropy growth due to the solid damageability as a specific characteristic of change in the structure organization. Following the tribo-fatigue ideas [2,3,4,51], the damageability is understood as any irreversible change in structure, continuum, shape, etc., of a deformable solid that leads to its limiting state. Although at plasticity modeling, the elasticity limit is not implicitly allowed for, the damageability at mechanical or contact fatigue proceeds in the course of linear elastic deformation. To describe it, we need a particular approach and must examine limiting fatigue characteristics of material. The below approach overcomes the above drawbacks.

## 3. Main Principles

**Figure 2:**
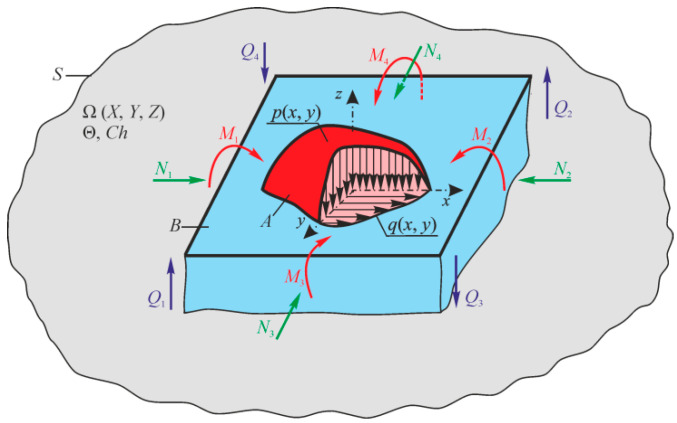
Mechanothermodynamical (MTD) system (**A**) denotes surface (contact) tractions; (**B**) denotes loaded body).

References [2,4,50] show that in the general case, an MTD system is given as a thermodynamic continuum where solids are distributed (scattered), interacting with each other and with the continuum. Figure 2 illustrates the continuum fragment of limited size Ω(X, Y, Z). The continuum with a temperature θ and a chemical composition *Ch* () has two solid elements (*A* and *B*) interacting in the contact zone S(x,  y,  z) that can move relatively to each other. Arbitrary mechanical loads perceived by one of them (by element *A*) in the *x*, *y*, *z* coordinate system are transformed into internal transverse forces *Q_x_, Q_y_, Q_z_*, longitudinal forces *N_x_, N_y_, N_z_*, as well as into bending moments *N_x_, N_y_, N_z_*. Element *B* is pressed to element *A* by the loads that are reduced to the distributed normal pressure *p*(*x*, *y*) and the tangential pressure *q*(*x*, *y*). The origin of the coordinates is shifted to the point of original contact *O* of the two elements (before deformation). We can easily notice that the elements *A* and *B* form the tribo-fatigue system [4], presented in Reference [2] as a friction pair (it consists of the element *A* without internal forces (*N_i_* = 0, *Q_i_* = 0, *M_i_* = 0, *i* = *x*, *y*, *z*) and of the element *B*). So, the tribo-fatigue system is the friction pair, in which, at least one of these elements perceives non-contact loads, and hence, it undergoes volumetric deformation. The advantage of such an MTD system is that the corresponding solutions reported in mechanics of deformable solids, contact mechanics, mechanics of tribo-fatigue systems (tribo-fatigue), and in tribology can be adopted to analyze the state of solids and the system components.

Now, it is the task to describe the MTD system energy state at mechanical and thermodynamic loads with the consideration of the environment influence. 

The energy state of any system is of interest. However, in relation to the MTD system, of importance is to examine its damageability, and as a consequence, conditions of reaching the limiting state. Of special interest is the analysis of the so-called translimiting or supercritical conditions [2].

According to References [2,3,4], we can formulate the main ideas, being the basis of the developed theory.

I. Bearing in mind that the MTD system elements perceive different loads: mechanical, thermal, and electrochemical, the traditional analysis of their damageability and limiting state at mechanical stresses or strains [53,54,55,56,57,58,59,60,61] can be a basis of studies, but it appears insufficient, and hence, ineffective. This means that the MTD system states must be analyzed using more general energy concepts.

II. Since mechanical, thermodynamic [62,63,64,65,66], and electrochemical loads specify the damageability of MTD system solids, we should use a generalized idea of its complex damage due to these loads when they act simultaneously. Such damage will be called any irreversible change in shape, size, volume, mass, composition, structure, continuity, and hence, physical-mechanical properties of system elements. It is a corresponding change in the functions of the system as a whole.

III. Four particular phenomena: mechanical fatigue, friction, and wear, as well as thermodynamic and electrochemical processes, specify the complex damage onset and development. These phenomena are called particular ones in the sense that each of them can be implemented as independent and individual. This leads to the corresponding energy state and damageability by particular (individual) criteria.

IV. In the general case*,* all particular phenomena and MTD system processes develop simultaneously and in one zone. The MTD system states are attributed to not one of any of the above phenomena, but to their joint (collective) development, and consequently, to their interaction.

V. If all the energy $U_{\sum }$ supplied to the MTD system is responsible for its physical state, then the condition of its damageability is specified by the effective (dangerous) part $U_{\sum }^{eff}<<U_{\sum }$ spent for generation, motion, and interaction of irreversible damages.

VI. The effective energy $U_{\sum }^{eff}$ at volumetric deformation of solids can be given in the form of the function of three energy components: thermal $U_{T}^{eff}$, force $U_{n}^{eff}$, and frictional $U_{\tau }^{eff}$:

(25) $$U_{\sum }^{eff}=F_{\Lambda }\left(U_{T}^{eff},U_{n}^{eff},U_{\tau }^{eff}\right)\;,$$

where, $F_{\Lambda }$ considers the irreversible kinetic interaction of particular damage phenomena. The components $U_{T}^{eff},U_{n}^{eff},U_{\tau }^{eff}$ of the effective energy $U_{\sum }^{eff}$ do not possess the additivity property.

VII. We allow for the processes of electrochemical (in particular, corrosion) damage of solids by introducing the parameter 0 ≤ *D_ch_* ≤ 1 and study them as electrochemical damageability when acted upon by temperature (*D_T_*_(*ch*)_), stress (*D*_σ(*ch*)_), as well as by corrosion and friction (*D*_τ(*ch*)_). So, Function (25) assumes the form:

(26) $$U_{\sum }^{eff}=F_{\Lambda }\left(U_{T(ch)}^{eff},\;U_{n(ch)}^{eff},\;U_{\tau (ch)}^{eff}\right)\;.$$

VIII. The condition for the effective energy $U_{\sum }^{eff}$ to attain its limiting value—the critical quantity *U*_0_ in some area of limited size—in the dangerous volume of the MTD system, serves as the generalized criterion of the limiting (critical) state.

IX. It is considered that the energy *U*_0_ is a fundamental constant for a given material and must not depend on testing conditions, input energy types, or damage mechanisms.

X. The three-dimensional (3D) area $V_{ij}\subset V_{0}$ of a deformable solid ($V_{0}$ is the working volume) with the critical state of the material of its components at all its points is called the dangerous volume.

XI. In the general case, the limiting (critical) state of the MDT system is attained not because effective energy components grow, and hence, because irreversible damages at individual different-nature loads are accumulated, but because they interact dialectically. Their direction is characterized by the development of spontaneous hardening-softening of materials at the considered operating conditions. Thus, when Function (26) is taken into account, the hypothesis of the limiting (critical) state of the MTD system can be presented in the following general form:

(27) $$\Phi (u_{\sigma (ch)}^{eff},\;u_{\tau (ch)}^{eff},\;u_{T(ch)}^{eff},\;\Lambda_{n\backslash k\backslash l},\;m_{k},\;u_{0})=0,$$

where, the m_k_ k = 1, 2, …, are some characteristic properties (hardening-softening) of contacting materials, and the $\Lambda_{k\backslash l\backslash n}⋛$ 1 are the functions (parameters) of dialectic interactions of effective energies (irreversible damages) at different-nature loads. It means that at Λ_k_ > 1, the damageability increase is realized, at Λ_l_ < 1—its decrease, and at Λ_n_ = 1—its stable development. 

XII. When item III is taken into consideration, hypothesis (27) must be multi-criterion from the physical viewpoint, i.e., it must describe not only the states of the system as a whole, but its individual elements through different criteria of performance loss (wear, fatigue damage, pitting, corrosion damage, thermal damage, etc.). In particular cases, we can attain the corresponding limiting (critical) states through one or two, three, or several criteria at a time. 

XIII. Attaining the limiting state:

(28) $$u_{\Sigma }^{eff}=u_{0}$$

means that the MTD system completely loses its integrity, i.e., all of its functions. At the same time, its elements reach the critical damageability:

(29) $$0<\psi_{u}^{eff}=u_{\Sigma }^{eff}/u_{0}$$

(30) $$\psi_{u}^{eff}\left(\psi_{\sigma (ch)},\psi_{\tau (ch)},\psi_{T(ch)},\Lambda_{k\backslash l\backslash n},m_{k}\right)=1$$

XIV. If *t* = *t*_0_ is the time of the system onset and $T_{⊕}$ is the time when the system reaches the limiting (critical) state, then the failure time of its functions is consistent with the relative lifetime (longevity) $t/T_{⊕}=1$. But the system lifetime $T_{*}$ as a material object is longer than its lifetime as a whole ($T_{*}>>T_{⊕}$), since at $t>T_{⊕}$, the long process of its degradation–disintegration is realized when a great number of remains, pieces, fragments, etc., are formed. This process develops when acted upon by not only possible mechanical loads, but mainly by the environment, up to the moment when the system as a material object dies at *t* = T_*_. The system death is its complete disintegration into an infinitely large number of ultimately small particles (atoms). The below conditions describe the translimiting existence of the system as a gradually disintegrating material object:

(31) $$\psi_{u}^{eff}\to \infty ,$$

(32) $$d_{\psi }^{}\to 0,$$

where $d_{\psi }^{}$ is the average size of disintegration particles. The organic relationship $\psi_{u}^{eff}(d_{\psi })$ must exist between $\psi_{\Sigma }^{}$ and $d_{\psi }^{}$. Then, the condition for the system death is:

(33) $$t/T_{*}=1$$

XV. The disintegration particles of the “old system” are not destroyed and are spent to form and increase a number of “new systems”. This is the essence of the MTD system evolution hysteresis.

## 4. Damageability Energy Theory and Limiting States

First, we concretize Function (25).

For the effective energy to be determined, we will consider the work of internal forces in the elementary volume *dV* of tribo-fatigue systems (A, B in Figure 2). In the general case, we can write the differential of work of internal forces and the temperature *dT*_Σ_ with the consideration of the disclosure rule of the biscalar product of the stress and strain tensors σ and ε:

(34) $$\begin{array}{l} du=\sigma_{ij}\cdot \cdot d\varepsilon_{ij}+kdT_{\Sigma }=\left(\begin{array}{ccc} \sigma_{xx} & \sigma_{xy} & \sigma_{xz}\\ \sigma_{yx} & \sigma_{yy} & \sigma_{yz}\\ \sigma_{zx} & \sigma_{zy} & \sigma_{zz} \end{array}\right)\cdot \cdot \left(\begin{array}{ccc} d\varepsilon_{xx} & d\varepsilon_{xy} & d\varepsilon_{xz}\\ d\varepsilon_{yx} & d\varepsilon_{yy} & d\varepsilon_{yz}\\ d\varepsilon_{zx} & d\gamma_{zy} & d\varepsilon_{zz} \end{array}\right)+\\ +kdT_{\Sigma }\;=\sigma_{xx}d\varepsilon_{xx}+\sigma_{yy}d\varepsilon_{yy}+\sigma_{zz}d\varepsilon_{zz}+\sigma_{xy}d\varepsilon_{xy}+\sigma_{xz}d\varepsilon_{xz}+\sigma_{yz}d\varepsilon_{yz}+kdT_{\Sigma }\;; \end{array}$$

where *k* is the Boltzmann constant.

We will proceed from the fact that in the general case, according to References [2,4], normal and shear stresses, which cause the processes of shear (due to friction) and tear (due to tension-compression), play a decisive role in forming wear-fatigue damage. 

In this case, it makes sense to divide the tensor σ into two parts: σ_τ_ is the friction-shear stress tensor, or, briefly, the shear tensor, σ*_n_* is the normal stress tensor (tension-compression), or, briefly, the tear tensor. In Equation (28), we will distinguish the tear part σ*_n_* and the shear part σ_τ_ of the tensor σ as:

(35) $$\begin{array}{l} du=\sigma_{ij}^{\left(V,\;W\right)}\cdot \cdot d\varepsilon_{ij}^{\left(V,\;W\right)}+kdT_{\Sigma }=\left(\sigma_{n}{}^{\left(V,\;W\right)}+\sigma_{\tau }{}^{\left(V,\;W\right)}\right)\cdot \cdot d\varepsilon_{ij}^{\left(V,\;W\right)}+kdT_{\Sigma }=\\ =\sigma_{n}{}^{\left(V,\;W\right)}\cdot \cdot d\varepsilon_{ij}^{\left(V,\;W\right)}+\sigma_{\tau }{}^{\left(V,\;W\right)}\cdot \cdot d\varepsilon_{ij}^{\left(V,\;W\right)}+kdT_{\Sigma }.=du_{n}+du_{\tau }+du_{T}. \end{array}$$

In accordance with items III and IV, we must present the tensors σ*_ij_* and ε*_ij_* as follows:

(36) $$\begin{array}{l} \sigma_{ij}^{}=\sigma_{ij}^{\left(V,\;W\right)}=\sigma_{ij}^{}\left(\sigma_{ij}^{\left(V\right)},\sigma_{ij}^{\left(W\right)}\right)\;,\\ \varepsilon_{ij}^{}=\varepsilon_{ij}^{\left(V,\;W\right)}=\varepsilon_{ij}^{}\left(\varepsilon_{ij}^{\left(V\right)},\varepsilon_{ij}^{\left(W\right)}\right)\;. \end{array}$$

where, the volume loads (the general cases of 3D bending, torsion, tension-compression) give rise to the stress and strain tensors with the superscript *V* and the contact interaction of system elements to those with the superscript *W*. 

We can present Expression (35) with regard to (36) as follows:

(37) $$\begin{array}{l} du=\sigma_{ij}^{\left(V,\;W\right)}\cdot \cdot d\varepsilon_{ij}^{\left(V,\;W\right)}+kdT_{\Sigma }=\left(\sigma_{n}{}^{\left(V,\;W\right)}+\sigma_{\tau }{}^{\left(V,\;W\right)}\right)\cdot \cdot d\varepsilon_{ij}^{\left(V,\;W\right)}+kdT_{\Sigma }=\\ =\sigma_{n}{}^{\left(V,\;W\right)}\cdot \cdot d\varepsilon_{ij}^{\left(V,\;W\right)}+\sigma_{\tau }{}^{\left(V,\;W\right)}\cdot \cdot d\varepsilon_{ij}^{\left(V,\;W\right)}+kdT_{\Sigma }\cdot =du_{n}+du_{\tau }+du_{T}. \end{array}$$

When there is a linear relationship between stresses and strains, Expression (36) will assume the form:

(38) $$\sigma_{ij}^{}=\sigma_{ij}^{\left(V,\;W\right)}=\sigma_{ij}^{\left(V\right)}+\sigma_{ij}^{\left(W\right)}=\left(\begin{array}{ccc} \sigma_{xx}^{\left(V\right)}+\sigma_{xx}^{\left(W\right)} & \sigma_{xy}^{\left(V\right)}+\sigma_{xy}^{\left(W\right)} & \sigma_{xz}^{\left(V\right)}+\sigma_{xz}^{\left(W\right)}\\ \sigma_{yx}^{\left(V\right)}+\sigma_{yx}^{\left(W\right)} & \sigma_{yy}^{\left(V\right)}+\sigma_{yy}^{\left(W\right)} & \sigma_{yz}^{\left(V\right)}+\sigma_{yz}^{\left(W\right)}\\ \sigma_{zx}^{\left(V\right)}+\sigma_{zx}^{\left(W\right)} & \sigma_{zy}^{\left(V\right)}+\sigma_{zy}^{\left(W\right)} & \sigma_{zz}^{\left(V\right)}+\sigma_{zz}^{\left(W\right)} \end{array}\right),$$

(39) $$\varepsilon_{ij}^{}=\varepsilon_{ij}^{\left(V,\;W\right)}=\varepsilon_{ij}^{\left(V\right)}+\varepsilon_{ij}^{\left(W\right)}=\left(\begin{array}{ccc} \varepsilon_{xx}^{\left(V\right)}+\varepsilon_{xx}^{\left(W\right)} & \varepsilon_{xy}^{\left(V\right)}+\varepsilon_{xy}^{\left(W\right)} & \varepsilon_{xz}^{\left(V\right)}+\varepsilon_{xz}^{\left(W\right)}\\ \varepsilon_{yx}^{\left(V\right)}+\varepsilon_{yx}^{\left(W\right)} & \varepsilon_{yy}^{\left(V\right)}+\varepsilon_{yy}^{\left(W\right)} & \varepsilon_{yz}^{\left(V\right)}+\varepsilon_{yz}^{\left(W\right)}\\ \varepsilon_{zx}^{\left(V\right)}+\varepsilon_{zx}^{\left(W\right)} & \varepsilon_{zy}^{\left(V\right)}+\varepsilon_{zy}^{\left(W\right)} & \varepsilon_{zz}^{\left(V\right)}+\varepsilon_{zz}^{\left(W\right)} \end{array}\right),$$

And Expression (37) will be as follows:

(40) $$\begin{array}{c} du=u=\frac{1}{2}\sigma_{ij}\cdot \cdot \varepsilon_{ij}+kT_{\Sigma }=\frac{1}{2}\left(\sigma_{ij}^{\left(V\right)}+\sigma_{ij}^{\left(W\right)}\right)\cdot \cdot \left(\varepsilon_{ij}^{\left(V\right)}+\varepsilon_{ij}^{\left(W\right)}\right)+\\ +kT_{\Sigma }=\frac{1}{2}\left[\left(\sigma_{n}^{\left(V\right)}+\sigma_{n}^{\left(W\right)}\right)+\left(\sigma_{\tau }^{\left(V\right)}+\sigma_{\tau }^{\left(W\right)}\right)\right]\cdot \cdot \left(\varepsilon_{ij}^{\left(V\right)}+\varepsilon_{ij}^{\left(W\right)}\right)+kT_{\Sigma }=\\ =\frac{1}{2}\left[\begin{array}{l} \left(\begin{array}{ccc} \sigma_{xx}^{\left(V\right)}+\sigma_{xx}^{\left(W\right)} & 0 & 0\\ 0 & \sigma_{yy}^{\left(V\right)}+\sigma_{yy}^{\left(W\right)} & 0\\ 0 & 0 & \sigma_{zz}^{\left(V\right)}+\sigma_{zz}^{\left(W\right)} \end{array}\right)+\\ +\left(\begin{array}{ccc} 0 & \sigma_{xy}^{\left(V\right)}+\sigma_{xy}^{\left(W\right)} & \sigma_{xz}^{\left(V\right)}+\sigma_{xz}^{\left(W\right)}\\ \sigma_{yx}^{\left(V\right)}+\sigma_{yx}^{\left(W\right)} & 0 & \sigma_{yz}^{\left(V\right)}+\sigma_{yz}^{\left(W\right)}\\ \sigma_{zx}^{\left(V\right)}+\sigma_{zx}^{\left(W\right)} & \sigma_{zy}^{\left(V\right)}+\sigma_{zy}^{\left(W\right)} & 0 \end{array}\right) \end{array}\right]\cdot \cdot \\ \cdot \cdot \left(\begin{array}{ccc} \varepsilon_{xx}^{\left(V\right)}+\varepsilon_{xx}^{\left(W\right)} & \varepsilon_{xy}^{\left(V\right)}+\varepsilon_{xy}^{\left(W\right)} & \varepsilon_{xz}^{\left(V\right)}+\varepsilon_{xz}^{\left(W\right)}\\ \varepsilon_{yx}^{\left(V\right)}+\varepsilon_{yx}^{\left(W\right)} & \varepsilon_{yy}^{\left(V\right)}+\varepsilon_{yy}^{\left(W\right)} & \varepsilon_{yz}^{\left(V\right)}+\varepsilon_{yz}^{\left(W\right)}\\ \varepsilon_{zx}^{\left(V\right)}+\varepsilon_{zx}^{\left(W\right)} & \varepsilon_{zy}^{\left(V\right)}+\varepsilon_{zy}^{\left(W\right)} & \varepsilon_{zz}^{\left(V\right)}+\varepsilon_{zz}^{\left(W\right)} \end{array}\right)+kT_{\Sigma }. \end{array}$$

From Expression (40), it is seen that the tear part σ*_n_* of the tensor σ is the sum of the tear parts of the tensors at the volumetric strain $\sigma_{n}^{\left(V\right)}$ and the surface load (friction) $\sigma_{n}^{\left(W\right)}$. The shear part σ_τ_ is the sum of the shear parts $\sigma_{\tau }^{\left(V\right)}$ and $\sigma_{\tau }^{\left(W\right)}$. This is the fundamental difference of the generalized approach to constructing a criterion for the MTD system limiting state. 

We will distinguish the effective part of total energy (Expression (40)) according to items V and VIII and References [2,3]. To do this, we will introduce the coefficients *A_n_*(*V*), *A*_τ_(*V*), and *A_T_*(*V*) of the corresponding dimension. The latter determine the absorbed energy fraction:

(41) $$du_{\Sigma }^{eff}=\Lambda_{M\backslash T}\left(V\right)\left\{\Lambda_{n\backslash \tau }\left(V\right)\left[A_{n}\left(V\right)\sigma_{n}\cdot \cdot d\varepsilon_{ij}+A_{\tau }\left(V\right)\sigma_{\tau }\cdot \cdot d\varepsilon_{ij}\right]+A_{T}\left(V\right)kdT_{\Sigma }\right\}$$

or

(42) $$du_{\Sigma }^{eff}=\Lambda_{M\backslash T}\left(V\right)\left\{\Lambda_{\tau \backslash n}\left(V\right)\left[A_{n}\left(V\right)du_{n}^{}+A_{\tau }\left(V\right)du_{\tau }^{}\right]+A_{T}\left(V\right)du_{T}^{}\right\}$$

where Λ*_М_*_\*T*_(*V*) and Λ_τ\__σ_(*V*) are the functions of interaction between different energies. The subscript τ\σ means the function Λ responsible for the interaction between the shear (τ) and tear (σ) components of the effective energy and the subscript *M*\*T*—the function Λ is responsible for the interaction between the mechanical (*M*) and thermal (*T*) parts of the effective energy. Generally speaking, the coefficients *A* can be different at different points of volume *V.* This fact allows one to take into account the environment inhomogeneity. 

Taking into consideration Expression (42), criteria (27) can be specified, not considering the environment influence:

(43) $$\Lambda_{M\backslash T}\left(V\right)\left\{\Lambda_{\tau \backslash n}\left(V\right)\left[du_{n}^{eff}+du_{\tau }^{eff}\right]+du_{T}^{eff}\right\}=u_{0}.$$

When there is a linear relationship between stresses and strains, Expressions (41) and (42) will be of the following form:

(44) $$u_{\Sigma }^{eff}=\Lambda_{M\backslash T}\left(V\right)\;\{\Lambda_{\tau \backslash n}\left(V\right)[\frac{1}{2}A_{n}\left(V\right)\;\sigma_{n}\cdot \cdot \varepsilon_{ij}+\frac{1}{2}A_{\tau }\left(V\right)\;\sigma_{\tau }\cdot \cdot \varepsilon_{ij}]+A_{T}\left(V\right)\;kT_{\Sigma }\},$$

or

(45) $$\begin{array}{cc} u_{\Sigma }^{eff}=\Lambda_{M\backslash T} & \left(V\right)\left\{\;\Lambda_{n\backslash \tau }\left(V\right)\;\left[A_{n}\left(V\right)u_{n}^{}\left(V\right)+A_{\tau }\left(V\right)u_{\tau }^{}\left(V\right)\right]+A_{T}\left(V\right)u_{n}^{}(V)\;\right\}=\\ & =\Lambda_{M\backslash T}\left(V\right)\left\{\;\Lambda_{n\backslash \tau }\left(V\right)\;\left[u_{n}^{eff}\left(V\right)+u_{\tau }^{eff}\left(V\right)\right]+u_{T}^{eff}(V)\right\}. \end{array}$$

With Expression (36) considered, criterion (43) can be presented as follows:

(46) $$u_{\Sigma }^{eff}=\{\;\left[u_{n}^{eff}(\sigma_{n}{}^{\left(V,\;W\right)},\varepsilon_{n}{}^{\left(V,\;W\right)})+u_{\tau }^{eff}(\sigma_{\tau }{}^{\left(V,\;W\right)},\varepsilon_{\tau }{}^{\left(V,\;W\right)})\right]\;\Lambda_{n\backslash \tau }+u_{T}^{eff}\}\;\Lambda_{T\backslash M}=u_{0}.$$

When time effects must be allowed for, criterion (46) will assume the form:

(47) $$\begin{array}{c} u_{\Sigma t}^{eff}=\int_{0}^{t}\{\;\left[u_{n}^{eff}(\sigma_{n}{}^{\left(V,\;W\right)},\varepsilon_{n}{}^{\left(V,\;W\right)},\;t)+u_{\tau }^{eff}(\sigma_{\tau }{}^{\left(V,\;W\right)},\varepsilon_{\tau }{}^{\left(V,\;W\right)},\;t)\right]\;\Lambda_{n\backslash \tau }(t)+\\ +u_{T}^{eff}(t)\}\;\Lambda_{T\backslash M}(t)\;dt=u_{0}. \end{array}$$

So, Expression (45) is the concretization of Equation (25) and Equation (46)—the concretization of criterion (27) when the environment influence is not taken into account.

Criterion (27) in the forms of Expressions (46) and (47) states: when the sum of interacting effective energy components at force, frictional, and thermal (thermodynamic) loads reaches a critical (limiting) quantity *u*_0_*,* the limiting (or critical) state of the MTD system (both as individual elements and the system as a whole) is implemented. Physically, it is attributed to many and different damage mechanisms.

Above, we noted the fundamental character of the parameter *u*_0_. Based on References [66,67,68,69,70,71,72,73,74,75,76,77,78], we will understand parameter *u*_0_ as the initial activation energy of the disintegration process*. u*_0_ approximately means both sublimation heat for metals and crystals with ionic bonds and thermal destruction activation energy for polymers:

$$u_{0}\approx u_{T}.$$

On the other hand, the quantity *u*_0_ is determined as the activation energy for mechanical fracture:

$$u_{0}\approx u_{M}.$$

Thus, the energy *u*_0_ can be a constant of a material:

(48) $$u_{0}\approx u_{M}\approx u_{T}=\mathrm{const}.$$

With the physical-mechanical and thermodynamic presentations of the damageability and fracture processes [67,68,70] taken into account, we write Expression (48) in the following form:

(49) $$u_{M}=s_{k}\frac{\sigma_{th}}{E}\frac{C_{a}}{\alpha_{V}}=u_{0}=kT_{S}\ln\frac{k\theta_{D}}{h}=u_{T},$$

where, *s_k_* is the reduction coefficient, σ*_th_* the theoretical strength, *E* the elasticity modulus, *C_a_* the atom heat capacity, α*_V_* the thermal expansion of the volume, *k* the Boltzmann constant, *T_S_* the melting point, θ*_D_* the Debye temperature, and *h* is the Planck constant. According to Expression (49), we can approximately assume [67]:

(50) $$u_{0}\approx \varepsilon_{*}\frac{C_{a}}{\alpha_{V}},$$

where ε_*_ ≈ 0.6 is the limiting strain of the interatomic bond. Calculations according to Expression (50) are not difficult. The methods for experimental determination of *u*_0_ have also been developed [68].

Equation (49) shows that *u*_0_ is the activation energy of a given material and is by the order of magnitude equal to 1…10 eV per one particle or molecule (~10^2^…10^3^ kJ/mol), i.e., it is close to the energy of interatomic bond rupture in the solid [71] and does not depend on a way of reaching rupture: mechanically, thermally, or by their simultaneous action. Reference [68] contains the tables of the *u*_0_ values for various materials.

Equation (49) gives a thermomechanical constant of a material [2]:

(51) $$\frac{\sigma_{th}}{T_{S}}=E\frac{\alpha_{V}k}{C_{a}}\ln\frac{k\theta_{D}}{h}=\theta_{\sigma }.$$

The constant θ_σ_ is the strength loss per *1 K*.

Criterion (46) is written in absolute values of physical parameters: effective and critical energy components. We can make this criterion dimensionless: it must be by divided by the quantity $u_{0}$. Criterion (46) is presented in terms of irreversible (effective) damage:

(52) $$\psi_{u}^{eff}=\frac{u_{\Sigma }^{eff}}{u_{0}}=1.$$

The local (at the point) energy damageability measure $\psi_{u}^{eff}$ is within the range:

(53) $$0\leq \psi_{u}^{eff}\leq 1,$$

or in expanded form:

(54) $$\begin{array}{c} 0\leq \psi_{u}^{eff}=\\ =\frac{\Lambda_{T\backslash M}}{u_{0}}\{\;\left[u_{n}^{eff}(\sigma_{n}{}^{\left(V,\;W\right)},\varepsilon_{n}{}^{\left(V,\;W\right)})+u_{\tau }^{eff}(\sigma_{\tau }{}^{\left(V,\;W\right)},\varepsilon_{\tau }{}^{\left(V,\;W\right)})\right]\;\Lambda_{n\backslash \tau }+u_{T}^{eff}\}\leq 1. \end{array}$$

According to Expression (54), we can determine particular energy damageability measures

(55) $$0\leq \psi_{n}^{eff}=\frac{u_{n}^{eff}\left(\sigma_{n}^{\left(V,\;W\right)},\;\varepsilon_{n}^{\left(V,\;W\right)}\right)}{u_{0}}\leq 1,$$

(56) $$0\leq \psi_{\tau }^{eff}=\frac{u_{\tau }^{eff}\left(\sigma_{\tau }^{\left(V,\;W\right)},\;\varepsilon_{\tau }^{\left(V,\;W\right)}\right)}{u_{0}}\leq 1,$$

(57) $$0\leq \psi_{T}^{eff}=\frac{u_{T}^{eff}}{u_{0}}\leq 1,$$

at effective different energies determined by force (the subscript n), frictional (the subscript τ), and thermodynamic (the subscript *T*) loads, respectively. We can now write criterion (52) in dimensionless form:

(58) $$\psi_{u}^{eff}=\left[\left(\psi_{n}^{eff}+\psi_{\tau }^{eff}\right)\Lambda_{n\backslash \tau }+\psi_{T}^{eff}\right]\Lambda_{N\backslash T}=1.$$

Based on Expression (58), we can reach the MTD system limiting state at the sum of interacting damages (0<ψ<1) for mechanical and thermodynamic loads equal to 1. Criterion (46) in the form of Expression (58) finds convenient use because all damageability measures are dimensionless and are within 0≤ψ≤1.

Since we cannot describe and predict exactly numerous and innumerable interactions between physical damages of many-type dislocation, vacancy, non-elastic deformation, etc., the analysis of the MTD system must use the concept of interaction between dangerous volumes [2] that contain a real complex of damages (defects as a result of the action of the corresponding stress/strain fields). By the statistical model of a deformable solid with a dangerous volume [71,72], such a volume of a solid must depend on its geometric parameters responsible for the working volume $V_{0}$, on the parameters of the distribution functions of $p(\sigma_{-1})$ and p(σ) of the durability limit $\sigma_{-1}$ and the effective stresses σ, considering both the effective stress probabilities *P* and $\gamma_{0}$, as well as the effective stress gradients $G_{\sigma }$:

(59) $$V_{P\gamma }=F_{V}\left[p(\sigma_{-1}),\;p(\sigma ),\;G_{\sigma },\;V_{0},\;P,\;\gamma_{0},\;\vartheta_{V}\right].$$

where, $\vartheta_{V}$ describes the influence of the shape of a body on the durability limit and the schemes of its loading in fatigue tests. 

The dangerous volume can then be taken as the equivalent of the damage complex*,* since its value is proportional, in particular to the value of effective stresses, and hence, to the number (concentrations) of defects (damages).

As follows from Expression (59), the boundary between dangerous and safe volumes is generally blurred and probabilistic in nature. By increasing the damage probability *P* of the solid, the dangerous volume $V_{P_{\gamma }}$ grows. At a given *P* value, the volume can vary depending on the confidence probability $\gamma_{0}$. It means that at *P* = const:

(60) $$V_{P_{\gamma \;\min}}\leq V_{P_{\gamma }}\leq V_{P_{\gamma \;\max}},$$

if $\gamma_{\min}\leq \gamma_{0}\leq \gamma_{\max}$. Here, $\gamma_{\min},\gamma_{\max}$ form a permissible range. If $\gamma_{0}$ = const, then the dangerous volume will have a single value associated with the damage probability *P*.

**Figure 3:**
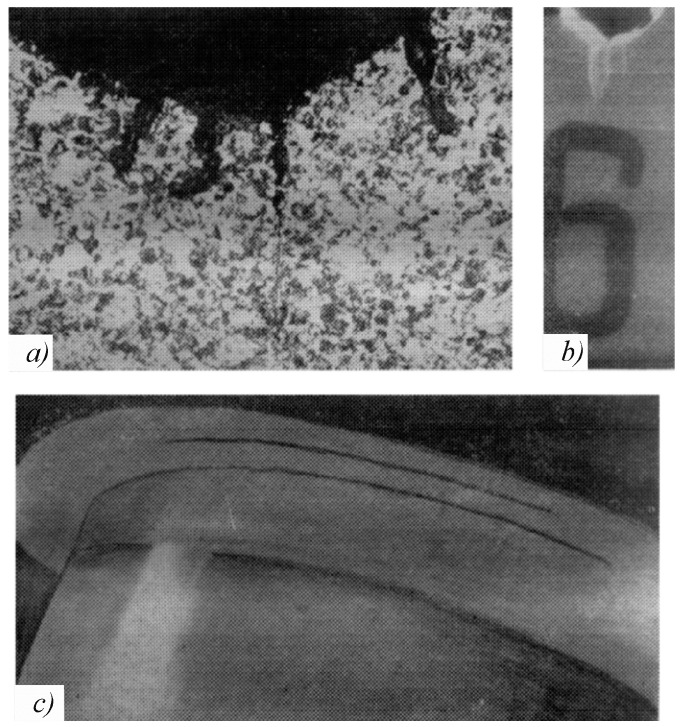
Microcracks in the zones of stress concentrators. (**a**) rounding-off radius *r* = 0.5 mm; (**b**) *r* = 2 mm;(**c**) *r* = 18 mm.

Not only the so-called smooth bodies, but also the elements with structural stress concentrators [71], are characterized by scattered damage within the dangerous volume. Figure 3 demonstrates several microcracks on the sharp cut (the rounding radius *r* = 0.5 mm, the theoretical stress concentration factor $\alpha_{n}$= 8, in Figure 3a) and on the flat cut (*r* = 2 mm, $\alpha_{n}$= 2.55, in Figure 3b) and also two fatigue cracks at a distance of 25 mm from each other at a fillet connection from the crankshaft journal to its web (*r* = 18 mm, $\alpha_{n}$= 3.2, in Figure 3c). The crankshaft journal diameter is 360 mm.

So, if in the case of the uniaxial stress state, the distribution of the stresses σ (*x*, *y*, *z*) in the *x*, *y*, *z* coordinates is known, then the dangerous volume is calculated by the formula:

(61) $$V_{P\gamma }=\underset{\sigma (x,y,z)>\sigma_{-1\min}}{∭}dxdydz,$$

where $\sigma_{-1\min}$–the lower boundary of the solid durability limit $\sigma_{-1}$ is such that if $\sigma_{-1}<\sigma_{-1\min}$, then *P* = 0.

Expression (61) yields the generalized condition for fatigue fracture in of the form:

(62) $$V_{P\gamma }>0$$

with some probability *P* at the confidence probability $\gamma_{0}$.

If

(63) $$V_{P\gamma }=0,$$

then fatigue fracture cannot occur physically (because in this case, $\sigma >\sigma_{-1\min}$); hence, Expression (63) is the generalized condition of non-fracture.

The methods to calculate dangerous volumes $V_{ij}$ for friction pairs and tribo-fatigue systems are developed similar to Expression (59)

(64) $$V_{ij}=V_{ij}\left(\sigma_{n}^{\left(V,\;W\right)},\;\sigma_{\tau }^{\left(V,\;W\right)},\;\sigma_{\lim}^{\left(V,\;W\right)},\;G_{\sigma_{ij}},\;V_{0},\;P,\;\gamma_{0}\right)$$

and outlined in References [4,71,72,73,74,75]. Here, $\sigma_{\lim}^{\left(V,\;W\right)}$ is the limiting stress by the assigned criterion of damage and fracture. 

Further, we can introduce the following dimensionless characteristics of damageability: integral energy damageability within the dangerous volume:

(65) $$\Psi_{u}^{eff}\left(V\right)=\underset{\psi_{u}^{eff}\left(dV\right)\geq 1}{∭}\frac{u_{\Sigma }^{eff}}{u_{0}}dV$$

and the average energy damageability (at each point of the dangerous volume):

(66) $${\bar{\Psi }}_{u}^{eff}\left(V\right)=\frac{1}{V_{u}}\underset{\psi_{u}^{eff}\left(dV\right)\geq 1}{∭}\frac{u_{\Sigma }^{eff}}{u_{0}}dV.$$

The time accumulation of energy damageability within the dangerous volume is governed by the formulas:

(67) $$\Psi_{u}^{eff}\left(V,t\right)=\int_{t}\underset{\psi_{u}^{eff}\left(dV\right)\geq 1}{∭}\frac{u_{\Sigma }^{eff}}{u_{0}}dVdt$$

(68) $${\bar{\Psi }}_{u}^{eff}\left(V,t\right)=\int_{t}\frac{1}{V_{u}}\underset{\psi_{u}^{eff}\left(dV\right)\geq 1}{∭}\frac{u_{\Sigma }^{eff}}{u_{0}}dVdt.$$

Based on Expressions (63)–(68), the MTD system damageability can be described and analyzed with the adoption of the most general concepts—the energy concepts allowing for the influence of numerous and different factors taken into account by Expression (59), including the scale effect*,* i.e., the changes in the size and shape (mass) of system elements.

In References [2,77], the function $\Lambda_{k\backslash l\backslash n}$ for damage interactions in the MTD system is determined by the effective energy ratio parameters:

(69) $$\Lambda_{n\backslash k\backslash l}=\Lambda_{n\backslash k\backslash l}\left(\rho_{M\backslash T},\rho_{n\backslash \tau }\right)⋛1,$$

(70) $$\rho_{n\backslash \tau }=u_{\tau }^{eff}/u_{n}^{eff},\;\rho_{M\backslash T}=u_{M}^{eff}/u_{T}^{eff}.$$

The quantities Λ calculated by Expression (69) describe how the load parameter ratio affects the character and direction of interaction of irreversible damages [2,3,4]. If Λ >1, then the system is self-softening, since, when hardening–softening phenomena are in balance, softening processes are dominant. If Λ *<1*, then the system is self-hardening, since, when hardening–softening phenomena are in balance, hardening processes are dominant. At Λ =1, the system is stable. The spontaneous hardening–softening phenomena are in balance. A particular article will deal with a general analysis of damage interactions in MTD systems because of its fundamental importance.

After criterion (27) has been basically formalized, the action of electrochemical loads (damages) should be taken into consideration in accordance with item VII. We must immediately emphasize that in the strict mechanothermodynamical statement, it is difficult to do this: when the environment interacts with a deformable solid, electrochemical reactions are very diverse, complex and insufficiently studied. That is why the approach proposed in References [2,3] was adopted: we introduced the simplification, according to which the damage of solids in the environment is determined by corrosion–electrochemical processes. In addition, the hypothesis is put forward, following which, the effective energy of corrosion–electrochemical damage is proportional to the square of the corrosion speed, i.e.,

(71) $$u_{ch}^{eff}\;\sim \;v_{ch}^{2}.$$

If, in accordance with item VII, $0\leq D_{ch}\leq 1$ is the parameter of corrosion–electrochemical damage of the solid, then from References [2,4,76], criterion (26) considering its shape will be of the form:

(72) $$\Lambda_{M\backslash T}\left[\left(\frac{u_{n}^{eff}\left(\sigma_{n}^{\left(V,W\right)},\;\varepsilon_{n}^{\left(V,W\right)}\right)}{u_{0}\left(1-D_{n}\right)}+\frac{u_{\tau }^{eff}\left(\sigma_{\tau }^{\left(V,W\right)},\;\varepsilon_{\tau }^{\left(V,W\right)}\right)}{u_{0}\left(1-D_{\tau }\right)}\right)\Lambda_{n\backslash \tau }+\frac{u_{T}^{eff}}{u_{0}\left(1-D_{T}\right)}\right]=1,\;\Lambda ⋛1$$

where

(73) $$0\leq \frac{u_{n}^{eff}\left(\sigma_{n}^{\left(V,W\right)},\;\varepsilon_{n}^{\left(V,W\right)}\right)}{u_{0}\left(1-D_{n}\right)}=\psi_{n(ch)}^{eff}\leq 1,$$

(74) $$0\leq \frac{u_{\tau }^{eff}\left(\sigma_{\tau }^{\left(V,W\right)},\;\varepsilon_{\tau }^{\left(V,W\right)}\right)}{u_{0}\left(1-D_{\tau }\right)}=\psi_{\tau (ch)}^{eff}\leq 1,$$

(75) $$0\leq \frac{u_{T}^{eff}}{u_{0}\left(1-D_{T}\right)}=\psi_{T(ch)}^{eff}\leq 1,$$

(76) $$1-D_{T}=b_{e(T)}{\left(\frac{v_{ch}}{v_{ch(T)}}\right)}^{m_{v(T)}};\;1-D_{n}=b_{e(n)}{\left(\frac{v_{ch}}{v_{ch(n)}}\right)}^{m_{v(n)}};$$

where *v_ch_* is the corrosion speed in this environment, *v_ch_*_(*T*)_, *v_ch_*_(σ)_, *v_ch_*_(τ)_ is the corrosion speed in the same environment at thermal, force, and friction loads respectively, the *b_e_*’s are the coefficients responsible for corrosive erosion processes, the $M_{V(•)}$’s are the parameters responsible for the electrochemical activity of materials at force (the subscript σ), friction (the subscript τ), and thermodynamic (the subscript *T*) loads, wherein $M_{V(•)}=2/A_{ch}$ and the parameter $A_{ch}⋛$ 1.

In Reference [76], we can find other methods for assessment of the parameter $D_{ch}$.

As seen, Equation (72) is the specification of criterion (27). According to this criterion, the limiting state of the MTD system is reached when the sum of dialectically interacting irreversible damages at force, friction, and thermodynamic loads (including electrochemical damage when acted upon by stress, friction, temperature) becomes equal to unity.

We consider the particular case: in Expression (46), it is assumed that *A*_σ_(*V*) = *A*_σ_ = const, *A*_τ_(*V*) = *A*_τ_ = const, *A_T_*(*V*) = *A_T_* = const, *A*_τ\__σ_(*V*) = *A*_τ\__σ_ = const, and *A_M_*_\*T*_(*V*) = *A_M_*_\*T*_ = const.

Firstly, the stress state is induced by volume deformation, for which all stress tensor components, with the exception of one component σ (one-dimensional tension–compression, pure bending), can be neglected. Secondly, the stress state is induced by surface friction, for which all stress tensor components, with the exception of one component $\tau_{w}$, can be neglected. Expression (40) then assumes the form:

$$\Lambda_{M\backslash T}\left[\Lambda_{\tau \backslash n}\left(A_{\sigma }\sigma^{2}+A_{\tau }\tau^{2}\right)+A_{T}T_{\Sigma }\right]=u_{0},$$

or in accordance with Expression (72):

(77) $$\Lambda_{M\backslash T}\left[\frac{a_{T}}{1-D_{T}}T_{\Sigma }+\Lambda_{n\text{\textbackslash{}}\tau }\left(\frac{a_{n}}{1-D_{n}}\sigma^{2}+\frac{a_{\tau }}{1-D_{\tau }}\tau_{w}^{2}\right)\right]=u_{0},\;\Lambda ⋛1$$

where $\frac{a_{\sigma }}{1-D_{\sigma }}=A_{\sigma }$, $\frac{a_{\tau }}{1-D_{\tau }}=A_{\tau }$, $\frac{a_{T}}{1-D_{T}}=A_{n}$.

Equation (77) is thus the simplest form of the energy criterion of the limiting state. Nevertheless, it is of great practical importance [2]. 

If the electrochemical influence of the environment is absent ($D_{ch}$ = 0), then:

(78) $$u_{\Sigma }^{eff}=\Lambda_{M\backslash T}\left[a_{T}T_{\Sigma }+\Lambda_{\tau \text{\textbackslash{}}n}\left(a_{\sigma }\sigma^{2}+a_{\tau }\tau_{w}^{2}\right)\right]=u_{0}\;,\;\Lambda ⋛1.$$

Equation (78) is the simplest form of the energy criterion of the limiting state and is of great practical importance [2,76,77,78,79,80,81]. In particular, it is used to develop methods for assessment of $a_{T},a_{\sigma },a_{\tau }$. In fact, at $\Lambda_{M\backslash T}=\Lambda_{\tau \text{\textbackslash{}}n}=1$, the boundary conditions are:

(79) $$\begin{array}{l} T_{\Sigma }=0\;,\tau_{w}=0:\;a_{n}\sigma_{d}^{2}=u_{0}\;,\;a_{n}=u_{0}/\sigma_{d}^{2}\;;\\ T_{\Sigma }=0\;,\sigma =0:\;a_{\tau }\tau_{d}^{2}=u_{0}\;,\;a_{\tau }=u_{0}/\tau_{d}^{2}\;;\\ \sigma =0\;,\tau_{w}=0:\;a_{T}\sigma_{d}^{}=u_{0}\;,\;a_{T}=u_{0}/T_{d}^{}\;, \end{array}\}$$

where $\sigma_{d},\tau_{d}$ are the force and friction limiting stresses as *T* → 0 and are called the (mechanical) destruction limits, and *T_d_* is the destruction temperature (when σ = 0, $\tau_{w}$ = 0) or the thermal destruction limit. 

The effective (“dangerous”) part of total strain energy can also be determined from the following physical considerations. The strain energy flux *u*, generated in the material sample at its cyclic strain (ε = ε_max_ sinω*t*) in the homogeneous (linear) stress state, is assumed to be similar to the light flux. In fact, it is continuously excited when the loading cycle is repeated with the speed ω1/λ. It can be considered as a wave of length λ. Some part of the energy *u* generated in such a way can be absorbed by material atoms and structural formations, which results in material damage. We denote the absorbed part of the energy by *u^eff^*. The generated energy *u* is then equal to:

(80) $$u=u^{eff}+u_{cons}$$

where, $u_{cons}$ is the non-absorbed part of the generated energy *u*. In this case, it is called the conservative part.

If the analogy of light and energy strain is valid, then the strain absorption law may be similar to Bouguer’s light absorption law. Consequently, the equation, linking the energy *u*_cons_ passed through the deformed material volume *V* and the generated energy *u,* has the form:

(81) $$u_{cons}=u\exp\left(-\chi_{\varepsilon }V\right),$$

or, by Lambert, in differential form:

(82) $$\frac{du}{u}=-\chi_{\varepsilon }V.$$

Here, as in Bourguer–Lambert’s equation, the coefficient χ_ε_ independent of *u* is the energy absorption parameter. 

Taking into account Equations (81) and (80), we obtain the strain energy absorption law:

(83) $$u^{eff}=u\left[1-\exp\left(-\chi_{\varepsilon }V\right)\right],$$

and hence, if *u* = 0 or *V* = 0, then *u^eff^* = 0. If *V* → ∞, it appears that according to Equation (81), *u*_cons_ = *u,* i.e., all input energy is dissipated within such a volume. 

Physically, the strain energy absorption process occurs due to many phenomena:

– Electron transition in absorbing atoms from lower to higher energy levels (quantum theory).
– Generation and development of dislocation structures (dislocation theory).
– Emergence of II and III order residual strains (stresses) (elasticity theory).
– Formation and development of any imperfections (defects) of material composition and structure: point, planar, and spatial (physical materials science).
– Hardening–softening phenomena (including strain aging) developing in time (fatigue theory).
– Changes in (internal) tribo-fatigue entropy (wear-fatigue damage mechanics [2]).

It should be noted that approach (83) can also be extended to friction, since any indenter drives a strain wave upstream in the thin surface layer of the solid. The indenter is pressed to the solid. Here, $\chi_{\gamma }$ is the energy absorption parameter and the subscript γ denotes the shear strain. Similarly, heat absorption in the deformable solid can also be considered. Finally, by introducing the dangerous volume $V=V_{P\gamma }$ into Equations (81)–(83), we can easily solve the problem of strain energy absorption in the non-uniform (including complex) stress state.

It should be noted that, although criterion (78) is special, it is fundamental and general in nature. Its general nature follows from the fact that this case takes into consideration all four particular phenomena responsible for the state of the MTD system (although simplified by the statement of the stress-strain state) in accordance with item III. Its fundamental nature is that here, as in the complete solution of Expression (46), $\Lambda_{n\backslash \tau }$ takes into account the interaction of effective mechanical energy components due to friction τ*_w_* and normal σ stresses, whereas $\Lambda_{M\backslash T}$ allows for the interaction of thermal and mechanical components of effective energy. The thermal component of the effective energy is determined by varying the total temperature *T*_Σ_ = *T*_2_ –*T*_1_ in the force contact zone induced by all heat sources, including the heat released during mechanical (spatial and surface) strain, structural changes, etc.

## 5. Mechanothermodynamical States

Within the framework of mechanothermodynamics, a special approach is being developed to assess the entropy in terms of a generalized energy state. Following this approach and Formula (77), the effective part of total energy (specific at some particular loads–force, temperature, etc.) directly spent for the damage production is defined by the experimentally found coefficients *A_l_* in Formulas (41), (42) and (77) [2,51,76].

(84) $$u_{l}^{eff}=A_{l}u_{l},$$

where the *u_l_*’s are the specific internal energies at tear (*u_n_*), shear (*u*_τ_), and thermal action (*u_T_*).

The total specific energy of an elementary volume and a rate of its change are then given as:

(85) $$u=\sum_{l}\left[\left(1-A_{l}\right)u_{l}+u_{l}^{eff}\right]\;;$$

(86) $$\frac{du}{dt}=\sum_{l}\left[\left(1-A_{l}\right)\frac{du_{l}}{dt}+\frac{du_{l}^{eff}}{dt}\right]$$

In addition, the Λ-functions are used to take account of a complex (non-additive) character of interactions between effective energies of different nature, expressed by Formula (42). This allows the total effective energy of the system to be assessed:

(87) $$u_{\Sigma }^{eff}=\Lambda_{\alpha }\left(u_{l}^{eff}\right)=\Lambda_{M\backslash T}\left(\Lambda_{\tau \backslash n},A_{l}u_{l}^{}\right)=\Lambda_{M\backslash T}\left\{\Lambda_{\tau \backslash n}\left[A_{n}u_{n}^{}+A_{\tau }u_{\tau }^{}\right]+A_{T}u_{T}^{}\right\},$$

where the Λ_α_’s are the possible combinations of interaction of effective energies (irreversible damages).

The specific feature of Λ-functions is such that:

(88) $$u_{\Sigma }^{eff}\;⋛\;u_{l}^{eff}$$

and hence,

(89) $$u_{\Sigma }^{eff}\;⋛\;\Sigma u$$

By using coefficients *A_l_* and Λ-functions, the energy interaction at different-nature loads can be found. Such interaction can give rise both to a sharp increase and a substantial decrease in the effective energy, resulting in damages and limiting states, in comparison to the energy calculated by the ordinary additivity model of type (17):

(90) $$u_{\Sigma }=\sum A_{l}u_{l}.$$

By taking account of Formula (87), the total effective energy of volume *V* and its accumulation in time have the form:

(91) $$U_{\Sigma }^{eff}=\int_{V}\rho u_{\Sigma }^{eff}\left(V\right)dV$$

and

(92) $$U_{\Sigma }^{eff}\left(t\right)=\int_{t}\int_{V}\rho u_{\Sigma }^{eff}\left(V,t\right)dVdt.$$

The principal moment of the mechanothermodynamical model is the account of the limiting state (limits of plasticity, strength, fatigue, etc.) according to item XIII (Section 3):

(93) $$u_{\Sigma }^{eff}=u_{0},$$

where *u*_0_ is the limiting density of the internal energy treated as the initial activation energy of the disintegration process.

A relationship between the current state (mechanical, thermomechanical, energy) of an elementary volume of a solid (medium) and its limiting state enables one to construct the parameter of local energy damageability: dimensionless:

(94) $$\psi_{u}^{eff}=\frac{u_{\Sigma }^{eff}}{u_{0}}$$

or dimensional:

(95) $$\psi_{u*\;}^{eff}=u_{\Sigma }^{eff}-u_{0}.$$

Local energy damageability (Equation (94) or (95)) is most general among the damageability parameters constructed in terms of different mechanical (thermomechanical) states φ [2,51,76]:

(96) $$\psi_{q}=\varphi_{q}/\varphi_{q}^{\left(*\lim\right)},$$

where ϕ = σ, ε, *u*; the σ’s are the stresses, the ε’s are the strains, *u* is the density of internal energy, the $\varphi_{q}^{\left(*\lim\right)}$’s are the limiting values of the state φ $q\in \{eqv,ij,i,S,{\;}_{ij}^{D},n,\tau ,\mathrm{int},$ $u,{\;}_{u}^{n},{\;}_{u}^{\tau },{\;}_{u}^{eff}\}$, *eqv* is the equivalent mechanical state, the *ij*’s are the components of the tensor ϕ, the *i*’s are the main components of the tensor ϕ, *S* and ${}_{ij}^{D}$ are the sphere and deviator parts of the tensor ϕ, *n* and τ are the normal and tangential components of the tensor ϕ, int is the intensity of ϕ, and *u* is the specific potential strain energy (internal energy density). The indices at *u* mean: ${}_{u}^{n}$ and ${}_{u}^{\tau }$ are the specific potential strain energy at tension–compression and shear, and ${}_{u}^{eff}$ is the effective specific potential strain energy.

We can build integral damageability measures on the basis of local measures (Equation (96)) using the model of a deformable solid with a dangerous volume (Equations (64)–(68)) [4,76]. 

The dangerous volume is called the spatial region of a loaded solid. At each point of a solid, the local damageability value is smaller than the limiting one [4,51,76]:

(97) $$V_{q}=\left\{\;dV/\varphi_{q}\geq \varphi_{q}^{\left(*\lim\right)},dV\subset V_{k}\right\}\;,$$

or

$$V_{q}=\left\{\;dV/\psi_{q}\geq 1,dV\subset V_{k}\right\}\;.$$

Dangerous volumes are calculated by the following general formula:

(98) $$V_{q}=\underset{\psi_{q}\left(V\right)\geq 1}{∭}dV.$$

The integral condition of damageability of a solid or a system can be written in the form:

(99) $$0<\omega_{q}=\frac{V_{q}}{V_{0}}<1,$$

where *V*_0_ is the working volume of the solid.

To analyze, at a time, dangerous volumes and local damageability distributed within them, we introduce the function of damageability of unit volume:

(100) $$d\Psi_{q}=\psi_{q}\left(V\right)dV.$$

The function of damageability of the entire volume *V* will then be as follows:

(101) $$\Psi_{q}^{}=\int_{\psi_{q}\geq 1}\psi_{q}\left(V\right)dV.$$

The simplest functions of damageability accumulation in time for unit volume and total volume will be have the following form, respectively:

(102) $$d\Psi_{q}^{\left(t\right)}=\int_{t}\psi_{q}\left(t\right)dt;$$

(103) $$\Psi_{q}^{\left(t\right)}=\int_{\psi_{q}\geq 1}\int_{t}\psi_{q}\left(V,t\right)dtdV.$$

The indices of volume-mean damageability

(104) $${\bar{\Psi }}_{q}^{\left(V\right)}=\frac{1}{V_{q}}\int_{\psi_{q}\geq 1}\psi_{q}\left(V\right)dV$$

and its accumulation in time can be used

(105) $${\bar{\Psi }}_{q}^{\left(V,t\right)}=\frac{1}{V_{q}}\int_{t}\int_{\psi_{q}\geq 1}\psi_{q}\left(V,t\right)dVdt.$$

The analysis of Formulas (94), (100) and (102) leads to the conclusion: conceptually, they are related to the entropy concept as a difference (or relations) between two states (configurations) of a system, the degree of its organization (chaotic state). In relation to damageability, such states are current and limiting. 

By using local energy damageability (Equation (94)), we construct specific (per unit mass) tribo-fatigue entropy (accurate constant):

(106) $$s_{TF}^{}=\psi_{u}^{eff}\left(\Lambda_{\alpha },A_{l},\sigma_{ij},T\right)=\underset{\Delta m->0}{\lim}A_{\psi }\frac{u_{\Sigma }^{eff}(\Delta m)}{u_{0}\Delta m},$$

or

(107) $$s_{TF}^{}=s_{TF*}^{}=\frac{\psi_{u*}^{eff}\left(\Lambda_{\alpha },A_{l},\sigma_{ij},T\right)}{T}=\frac{u_{\Sigma }^{eff}-u_{0}}{T}.$$

where $A_{\psi }$ is the dimensional parameter (J∙mol^–1^∙K^–1^).

On the basis of Expression (18) for entropy and Formulas (85) and (86), the local entropy and the rate of its change within an elementary volume will be:

(108) $$s=\frac{1}{T}\sum_{l}\left[\left(1-A_{l}\right)u_{l}\right]+s_{TF}^{}d\psi_{q}^{}=\psi_{q}^{}(V)dV$$

and

(109) $$\frac{ds}{dt}=\frac{1}{T}\sum_{l}\left[\left(1-A_{l}\right)\frac{du_{l}}{dt}\right]+\frac{ds_{TF}^{}}{dt}.$$

Formulas (108) and (109) show that unlike the thermomechanical model, the state indicators of the mechanothermodynamical system *u* and *s* are not equivalent. This is due to the fact that the calculation of the tribo-fatigue entropy *s_TF_* by Formula (106) is supplemented by the limiting state in the form of the limiting density of the internal energy *u*_0_.

The tribo-fatigue entropy *S_TF_* is calculated not within the total volume *V*, but only within its damaged part, i.e., within the energy effective dangerous volume $V_{u}^{eff}$:

(110) $$V_{u}^{eff}=\left\{\;dV/u_{\Sigma }^{eff}\geq u_{0},dV\subset V_{k}\right\}\;.$$

Based on Formulas (11), (106) and (110), the tribo-fatigue entropy of volume *V* will be:

(111) $$S_{TF}^{}=\int_{u_{\Sigma }^{eff}(V)\geq u_{0}}\rho s_{TF}\left(V\right)dV=\int_{u_{\Sigma }^{eff}(V)\geq u_{0}}\rho \psi_{u}^{eff}\left(V\right)dV,$$

where,

(112) $$\psi_{u}^{eff}(V)=\frac{u_{\Sigma }^{eff}(V)}{u_{0}}\;\mathrm{or}\;\psi_{u}^{eff}(V)=\frac{\psi_{u*}^{eff}(V)}{T}=\frac{u_{\Sigma }^{eff}(V)-u_{0}}{T(V)},$$

and its accumulation will be:

(113) $$S_{TF}^{}\left(t\right)=\int_{t}\int_{u_{\Sigma }^{eff}(V,t)\geq u_{0}}\rho s_{TF}\left(V,t\right)dVdt=\int_{t}\int_{u_{\Sigma }^{eff}(V,t)\geq u_{0}}\rho \psi_{u}^{eff}\left(V,t\right)dVdt,.$$

where,

(114) $$\psi_{u}^{eff}(V,t)=\frac{u_{\Sigma }^{eff}(V,t)}{u_{0}}\;\mathrm{or}\;\psi_{u}^{eff}(V,t)=\frac{\psi_{u*}^{eff}(V,t)}{T(V,t)}=\frac{u_{\Sigma }^{eff}(V,t)-u_{0}}{T(V,t)}.$$

We should emphasize the fundamental feature of tribo-fatigue total *S_TF_* and specific *s_TF_* entropies. So, a difference between two states can be assessed not only quantitatively (as thermomechanical entropy), but also qualitatively, because *s_TF_* is calculated through the limiting density of the internal energy *u*_0_. So, *s_TF_* and *S_TF_* allow us to answer how much the current state of a solid or a system is dangerous in comparison to limiting states. 

The total entropy and the rate of its change for a system solid with regard to Equations (111) and (113) assume the form:

(115) $$S=\int_{V}\frac{1}{T(V)}\sum_{l}\rho \left[\left(1-A_{l}(V)\right)u_{l}(V)\right]\;dV+S_{TF}^{}$$

and

(116) $$\frac{dS}{dt}=\int_{V}\frac{1}{T(V)}\sum_{l}\rho \left[\left(1-A_{l}(V)\right)\frac{du_{l}(V)}{dt}\right]\;dV+\frac{dS_{TF}^{}}{dt}$$

Based on Formulas (106)–(116), we can build the function of total entropy accumulation in time*:*

(117) $$\begin{array}{c} S\left(t\right)=\int_{t}\int_{V}\sum_{l}\rho s_{l}(V,t)dVdt+\int_{t}\int_{u_{\Sigma }^{eff}(V,t)\geq u_{0}}\rho s_{TF}(V,t)dVdt=\\ =\int_{t}\int_{V}\frac{1}{T(V,t)}\sum_{l}\rho \left[\left(1-A_{l}(V,t)\right)\frac{du_{l}(V,t)}{dt}\right]dVdt+\int_{t}\int_{u_{\Sigma }^{eff}(V,t)\geq u_{0}}\rho \psi_{u}^{eff}(V,t)dVdt. \end{array}$$

Practically, bearing in mind the limiting states of a solid or a system, models (115)–(117) can answer whether the current state is a qualitative jump in the system, i.e., whether the current state is close to the limiting (critical fatigue fracture entropy) one. A similar (dialectical as a matter of fact) qualitative transition differs from the bifurcation point in the ability to predict the system behavior after a transition on the basis of the analysis of *s_TF_* and *S_TF_*. Particular limiting states (limit of strength, mechanical or contact fatigue, etc.) enable for predicting the situation after passing the given point: principal changes in the system properties and behavior or the formation of a new system based on the previous one. 

An example can be non-linear deformation or generation of microcracks in the solid (or the system) that changes its strength and fatigue properties, and hence, its response to loads. In turn, formed macrocracks lead to local continuum violation—formation of new free surfaces (possibly, of new solids—destruction products), i.e., a new system.

It should be noted that models (115)–(117) were built using a traditional concept of entropy additivity (Equation (10)), although with the consideration of significant refinements. These models also contain reversible processes described by the entropy components *s_l_*, not yielding primary damages, and hence, the limiting states: the points of qualitative change in the system.

The assessment of the entropy state on the basis of the mechanothermodynamical model of a solid, which uses only tribo-fatigue entropy, is more advisable for a qualitative and quantitative analysis of evolution of systems passing through the states traditionally defined as bifurcation branches. In this case, Formulas (111)–(113) for entropy and their accumulation will be of the form:

(118) $$S=S_{TF}^{}=\int_{u_{\Sigma }^{eff}(V,t)\geq u_{0}}\rho s_{TF}\left(V\right)dV=\int_{u_{\Sigma }^{eff}(V,t)\geq u_{0}}\rho \psi_{u}^{eff}\left(V\right)dV,$$

and

(119) $$S\left(t\right)=S_{TF}^{}\left(t\right)=\int_{t}\int_{u_{\Sigma }^{eff}(V,t)\geq u_{0}}\rho s_{TF}\left(V\right)dVdt=\int_{t}\int_{u_{\Sigma }^{eff}(V,t)\geq u_{0}}\rho \psi_{u}^{eff}(V,t)dVdt.$$

To identify the points of qualitative change in the limiting states of solids (systems), we can use the indices of relative integral entropy and its accumulation using the concept of the integral condition of solid damageability (Equation (99)):

(120) $$\omega_{S}=\frac{S_{TF}}{V_{0}}=\frac{1}{V_{0}}\int_{u_{\Sigma }^{eff}(V,t)\geq u_{0}}\rho s_{TF}\left(V\right)dV;$$

(121) $$\omega_{S}\left(t\right)=\frac{S_{TF}\left(t\right)}{V_{0}}=\frac{1}{V_{0}}\int_{t}\int_{u_{\Sigma }^{eff}(V,t)\geq u_{0}}\rho s_{TF}\left(V\right)dVdt.$$

The values of *S_TF_*, *S_TF_* (*t*), ω*_S_*, ω*_S_*(*t*) can grow infinitely, allowing for not only describing the limiting states of type (93), but also different transmitting states. In essence, they “provide” a quantitative description of the entropy increase.

Now, based on Formulas (24), (115), (117) and (119), we construct generalized expressions for entropy, a rate of its change, and its accumulation in the MTD system consisting of a liquid (gas) medium of volume *V* and a solid of volume *V*_ψ_:

(122) $$\begin{array}{l} S=\int_{V_{}}\rho s_{T}dV+\int_{V_{\psi }}\sum_{l}\rho s_{l}dV_{\psi }+\int_{u_{\Sigma }^{eff}\geq u_{0}}\rho s_{TF}dV_{\psi }=\int_{V_{}}\frac{1}{T}\sigma_{ij}^{}\varepsilon_{ij}dV_{}+\int_{V_{}}\frac{1}{T}\rho qdV_{}+\\ +\int_{V_{}}\frac{1}{T}\rho \sum_{k}^{}\mu_{k}n_{k}\;dV_{}+\int_{V_{\psi }}\frac{1}{T}\sum_{k}\rho \left[\left(1-a_{k}\right)u_{k}\right]dV_{\psi }+\int_{u_{\Sigma }^{eff}\geq u_{0}}\rho \psi_{u}^{eff}dV_{\psi }; \end{array}$$

(123) $$\begin{array}{l} \frac{dS}{dt}=\int_{V}\rho \frac{ds_{T}}{dt}dV_{}+\int_{V_{\psi }}\sum_{l}\rho \frac{ds_{l}}{dt}dV_{\psi }+\int_{V_{\psi }}\rho \frac{ds_{TF}}{dt}dV_{\psi }=\int_{V}\frac{1}{T}\sigma_{ij}^{}\frac{d\varepsilon_{ij}}{dt}dV+\\ +\int_{V_{}}\frac{1}{T}\rho \frac{dq}{dt}dV+\int_{V_{}}\frac{1}{T}\rho \sum_{k}^{}\mu_{k}\frac{dn_{k}}{dt}\;dV_{}+\\ +\int_{V_{\psi }}\frac{1}{T}\sum_{k}\rho \left[\left(1-a_{k}\right)\frac{du_{k}}{dt}\right]dV_{\psi }+\int_{u_{\Sigma }^{eff}\geq u_{0}}\rho \frac{d\psi_{u}^{eff}}{dt}dV_{\psi }; \end{array}$$

(124) $$\begin{array}{l} S(t)=\int_{t}\int_{V}\rho s_{T}dV\;dt+\int_{t}\int_{V_{\psi }}\sum_{l}\rho s_{l}dV_{\psi }dt+\int_{t}\int_{u_{\Sigma }^{eff}\geq u_{0}}\rho s_{TF}dV_{\psi }dt=\int_{t}\int_{V}\frac{1}{T}\sigma_{ij}^{}\varepsilon_{ij}dVdt+\\ +\int_{t}\int_{V}\frac{1}{T}\rho qdVdt+\int_{t}\int_{V}\frac{1}{T}\rho \sum_{k}^{}\mu_{k}n_{k}\;dVdt+\int_{t}\int_{V\psi }\frac{1}{T}\sum_{l}^{}\rho \left[\left(1-a_{l}\right)u_{l}\right]dV_{\psi }dt+\\ +\int_{t}\int_{u_{\Sigma }^{eff}\geq u_{0}}\rho \psi_{u}^{eff}dV_{\psi }dt \end{array}$$

Similarly, we can build entropy state values for a system consisting of many media.

It should be noted that in Formulas (122)–(125), the interaction (contact) of two media, which can be complex in nature, is taken into account only implicitly in terms of medium state parameters (stress, strain, temperature). It is obvious that this is only the first step to a comprehensive (generalized) solution of the problem stated.

The simplified writing of Expression (123) for the entropy increment of the mechanothermodynamical system consisting of finite volumes *dV* and *dV*_ψ_ was presented in Reference [51] as follows:

(125) $$dS={\left(dS\right)}_{T}+{\left(d_{i}S\right)}_{TF}=\frac{dU+\Delta pdV}{T}-\frac{1}{T}\sum_{k}^{}\mu_{k}dN_{k}+\Psi_{u}^{eff}dV_{\psi }.$$

Expression (125) can also be presented in terms of specific quantities as:

(126) $$dS=\int_{V}\frac{\rho du+\rho dp}{T}dV-\int_{V}\frac{1}{T}\rho \sum_{k}^{}\mu_{k}dn_{k}\;dV+\int_{u_{\Sigma }^{eff}\geq u_{0}}\rho d\psi_{u}^{eff}dV_{\psi }$$

or on the basis of Expression (123):

(127) $$\frac{dS}{dt}=\int_{V}\frac{\sigma_{ij}^{}d\varepsilon_{ij}+\rho dq}{Tdt}dV-\int_{V}\frac{1}{T}\rho \sum_{k}^{}\mu_{k}\frac{dn_{k}}{dt}\;dV+\int_{u_{\Sigma }^{eff}\geq u_{0}}\rho \frac{d\psi_{u}^{eff}}{dt}dV_{\psi }.$$

In Formulas (111)–(113) for calculation of the tribo-fatigue entropy *S_TF_* and its accumulation *S_TF_*(*t*), the specific entropy *s_TF_* is assumed to be integrated in terms of the damageable region of the solid alone—the dangerous volume. However, the influence of undamageable regions can also be allowed for by integrating *S_TF_* within the total volume:

(128) $$S_{TF}^{}=\int_{V}\rho s_{TF}\left(V\right)dV=\int_{V}\rho \psi_{u}^{eff}\left(V\right)dV;$$

(129) $$S_{TF}^{}\left(t\right)=\int_{t}\int_{V}\rho s_{TF}\left(V,t\right)dVdt=\int_{t}\int_{V}\rho \psi_{u}^{eff}\left(V,t\right)dVdt,$$

where,

(130) $$\psi_{u}^{eff}=\left\{ \begin{array}{l} \frac{u_{\Sigma }^{eff}\left(V,t\right)}{u_{0}}\geq 1,\;\mathrm{if}\;u_{\Sigma }^{eff}\geq u_{0};\\ \frac{u_{\Sigma }^{eff}\left(V,t\right)}{u_{0}}<1,\;\mathrm{if}\;u_{\Sigma }^{eff}<u_{0}, \end{array}\right.$$

or

(131) $$\psi_{u}^{eff}=\frac{\psi_{u*}^{eff}\left(V,t\right)}{T\left(V,t\right)}=\left\{ \begin{array}{l} \frac{u_{\Sigma }^{eff}\left(V,t\right)-u_{0}}{T\left(V,t\right)}\geq 0,\;\mathrm{if}\;u_{\Sigma }^{eff}\geq u_{0};\\ \frac{u_{\Sigma }^{eff}\left(V,t\right)-u_{0}}{T\left(V,t\right)}<0,\;\mathrm{if}\;u_{\Sigma }^{eff}<u_{0}. \end{array}\right.$$

Expression (131) shows that $\psi_{u}^{eff}$ < 0 is observed outside the dangerous volume (at $u_{\Sigma }^{eff}<u_{0}$). This means that the specific tribo-fatigue entropy *s_TF_* also appears to be negative (or less than unity for its alternative definition) outside the dangerous volume where the limiting state is not reached. Negative values of $\psi_{u}^{eff}$ and *s_TF_* can then be interpreted as the case where damageability is absent. In other words, the structure and/or properties of the solid are preserved.

The foregoing reports that the entropy additivity assumption is wrong in the general case for a system, consisting of a solid and a liquid (gas), where chemical reactions can occur. By analogy with the Λ-functions of interaction of different energies (Equation (179)), the functions of interaction of different entropies must be introduced by adding them to Expression (125) to determine total effective entropy:

(132) $$\begin{array}{l} dS_{total}^{eff}=\Lambda_{T\backslash TF}^{\left(S\right)}\left(dS_{T}+d_{i}S_{TF}\right)=\Lambda_{T\backslash TF}^{\left(S\right)}\left[\Lambda_{Q\backslash Ch}^{\left(S\right)}\left(dS_{T}^{Q}+dS_{Ch}^{Q}\right)+d_{i}S_{TF}\right]=\\ =\Lambda_{T\backslash TF}^{\left(S\right)}\left[\Lambda_{Q\backslash Ch}^{\left(S\right)}\left(\frac{dU+\Delta pdV}{T}-\frac{1}{T}\sum_{k}^{}\mu_{k}dN_{k}\right)+\Psi_{u}^{eff}dV_{\psi }\right], \end{array}$$

or

(133) $$\begin{array}{l} dS_{total}^{eff}=\Lambda_{T\backslash TF\backslash Ch}^{\left(S\right)}\left(dS_{T}+d_{i}S_{TF}\right)=\\ =\Lambda_{T\backslash TF\backslash Ch}^{\left(S\right)}\left[\frac{dU+\Delta pdV}{T}-\frac{1}{T}\sum_{k}^{}\mu_{k}dN_{k}+\Psi_{u}^{eff}dV_{\psi }\right], \end{array}$$

where the subscripts *Q* and *Ch* denote the thermodynamic and chemical entropy components.

Formulas (132)–(133) are supplemented by the generalized interaction functions $\Lambda_{T\backslash TF}^{\left(S\right)}$, $\Lambda_{Q\backslash Ch}^{\left(S\right)}$, and $\Lambda_{T\backslash TF\backslash Ch}^{\left(S\right)}$ in MTD systems. This means that the hypothesis about the thermodynamic and tribo-fatigue entropy additivity is not accepted. The corresponding interaction Λ-functions must be concretized and introduced into Equations (132)–(133).

## 6. Entropy Calculation under Simultaneous Contact and Non-Contact Loading

Consider the example of entropy calculation for the tribo-fatigue system consisting of friction pair with the elliptic contact of the ratio between smaller *b* and bigger *a* semi-axes *b/a* = 0.574. One of the elements of the friction pair is loaded by non-contact bending. An example of such an element is the shaft in the roller/shaft tribo-fatigue system.

In the case of the contact interaction over the elliptical area, the pressure is expressed as:

$$p_{}^{\left(n\right)}\left(x,y\right)=p_{0}^{\left(c\right)}\sqrt{\left(1-x^{2}/a^{2}-x^{2}/b^{2}\right),}$$

where $p_{0}^{\left(c\right)}$ is the maximum contact stress under the action of force $F_{c}$.

The entropy calculation system was based on the following initial data:

(134) $$\begin{array}{c} p_{0}^{\left(c\right)}={\sigma_{zz}^{\left(n\right)}\left(F_{c}\right)|}_{x=0,y=0,z=0}=\;2960\;\mathrm{MPa},\\ p_{0}^{\left(c,\lim\right)}=p_{0}\left(F_{c}^{\left(\lim\right)}\right)=888\;\mathrm{MPa}=0.3p_{0}^{\left(c\right)} \end{array}$$

where $p_{0}^{\left(c,\lim\right)}$ is the contact fatigue limit (maximum contact stress under the action of the limiting force $F_{c}^{\left(\lim\right)}$ obtained in the course of mechano-rolling fatigue tests described in References [1,2,3]. The criterion of the limiting state in these tests was the limiting approach of the axes in the tribo-fatigue system (100 μm). The test base was equal to 3⋅10^7^ cycles.

Calculations of the three-dimensional stress-strain state in the neighborhood of the elliptic contact for *b*/*a* = 0.574 [4] show that the maximum value of the strain energy *u* is related to the maximum contact pressure $p_{0}^{\left(c\right)}$ in the following way:

(135) $$u=\underset{dV}{\max}\left[u\left(F_{c}^{},\;dV\right)\right]=0.47p_{0}^{\left(c\right)}.$$

The limiting value of the strain energy $u_{}^{\left(lim\right)}$ under the action of the limiting force $F_{c}^{\left(\lim\right)}$ is:

(136) $$u_{}^{\left(lim\right)}=\underset{dV}{max}\left[u\left(F_{c}^{\left(\lim\right)},\;dV\right)\right]=0.47p_{0}^{\left(c,\lim\right)}.$$

In the calculations performed, maximum stresses $\sigma_{a}$ due to non-contact bending in the contact area were the following:

$$-0.34\leq \sigma_{a}/p_{0}^{\left(c\right)}\leq 0.34.$$

Tangential surface forces (friction force is directed along the major semi-axis of the contact ellipse) are:

$$p_{}^{\left(\tau \right)}\left(x,y\right)=-fp_{}^{\left(n\right)}\left(x,y\right)=-fp_{0}^{\left(c\right)}\sqrt{\left(1-x^{2}/a^{2}-x^{2}/b^{2}\right).}$$

**Figure 4:**
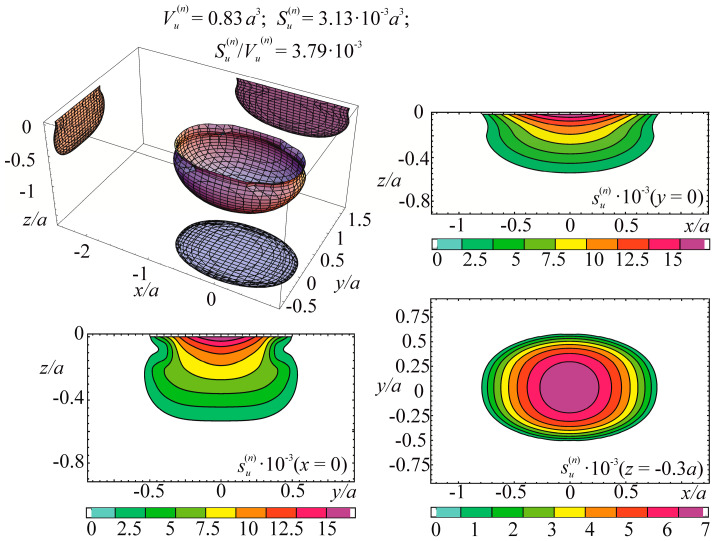
Energy dangerous volume and its sections, with specific entropy distributions for contact interaction without friction.

**Figure 5:**
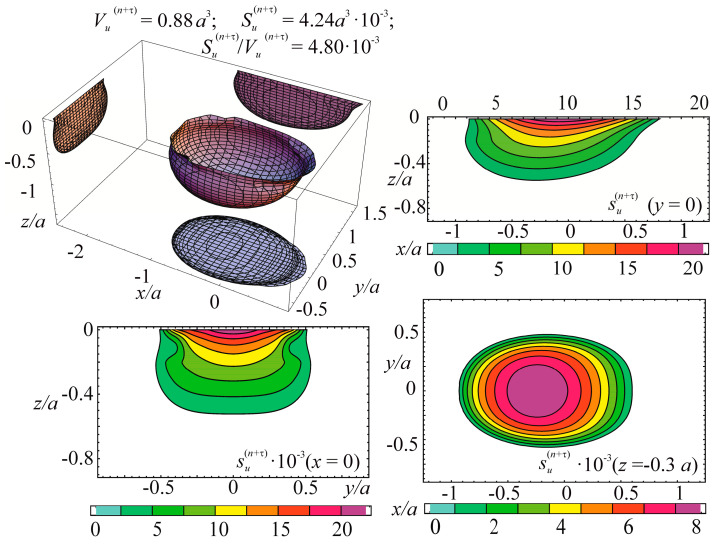
Energy dangerous volume and its sections, with specific entropy distributions for contact interaction with friction.

**Figure 6:**
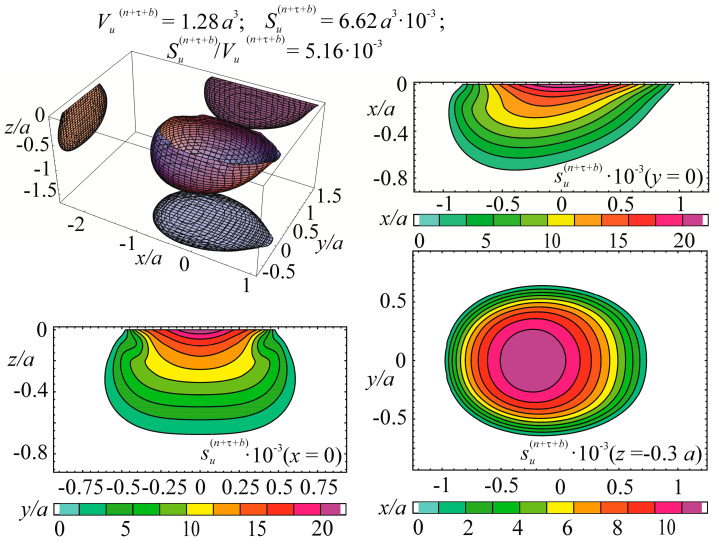
Energy dangerous volume and its sections, with specific entropy distributions for contact interaction with friction and tensile stresses $\sigma_{a}/p_{0}^{\left(c\right)}=0.34$ in the contact area caused by non-contact bending.

**Figure 7:**
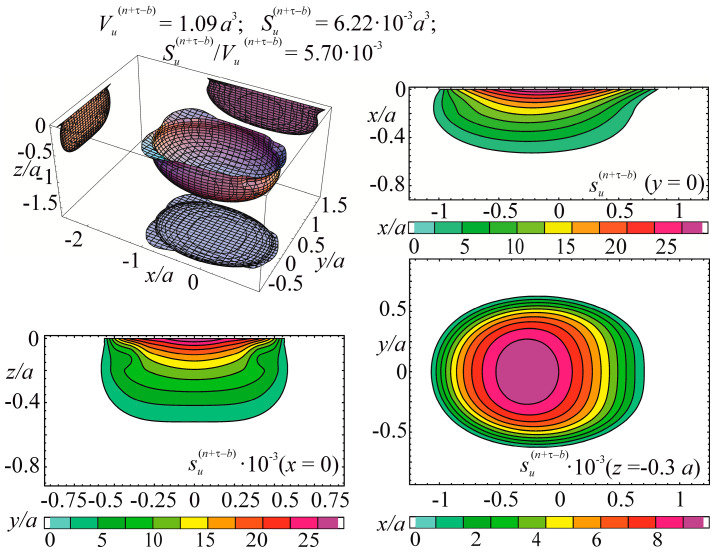
Energy dangerous volume and its sections, with specific entropy distributions for contact interaction with friction and compressive stresses $\sigma_{a}/p_{0}^{\left(c\right)}=-0.34$ in the contact area caused by non-contact bending.

The specific entropy distribution calculated according to Equation (112) shown in Figure 4, Figure 5, Figure 6 and Figure 7 can be considered to be the characteristic of the probability of appearance of local damages (initial cracks). The higher the specific entropy at a point of a dangerous volume, the greater the probability of initiation of damage (crack) at this point. The values of dangerous volume and entropy are the integral damageability indices (including a possible number of cracks and their sizes) of a solid or a system.

From Figure 4, Figure 5, Figure 6 and Figure 7 for $p_{0}^{}=p_{0}^{\left(c\right)}$ and the friction coefficient *f* = 0.2, the maximum specific entropy is in the center of the contact area. 

Under the joint action of contact pressure and tangential surface forces (friction) $s_{u}^{\left(n+\tau \right)}$, the maximum specific entropy increases by about 30% in comparison with the maximum specific entropy $s_{u}^{\left(n\right)}$. The joint action of contact pressure, friction, and tension due to bending increases $s_{u}^{\left(n+\tau +b\right)}$ by about 30% in comparison with $s_{u}^{\left(n\right)}$. At compressive bending, $s_{U}^{\left(n+\tau -b\right)}$ increases by about 60% in comparison with $s_{u}^{\left(n\right)}$. 

In case of frictional contact, the values of the dangerous volumes $V_{u}^{\left(n+\tau \right)}$, the entropy $S_{u}^{\left(n+\tau \right)}$, and the average entropy $S_{u}^{\left(n+\tau \right)}/V_{u}^{\left(n+\tau \right)}$, increase by about 6%, 35%, and 27%, as compared to $V_{u}^{\left(n\right)}$, $S_{u}^{\left(n\right)}$, and $S_{u}^{\left(n\right)}/V_{u}^{\left(n\right)}$, respectively.

**Figure 8:**
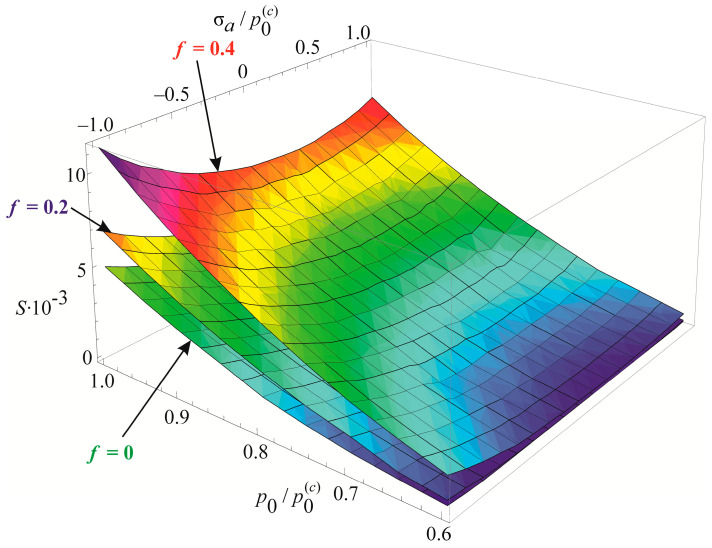
Entropy versus contact and non-contact stresses.

A more detailed analysis of the considered effects might be done using Figure 8. It shows a significant growth of entropy with increasing contact pressure, friction coefficient, and stresses caused by non-contact loads. The entropy increases almost at the same level for the same absolute values of tensile and compressive non-contact stresses. This effect may be due to the fact that the energy *u* attains positive values. 

The main conclusion of Figure 4, Figure 5, Figure 6, Figure 7 and Figure 8 is that not only friction, but also non-contact forces significantly change entropy characteristics in the neighborhood of the contact area.

Note that according to Expressions (95), (97) and (112), calculations were performed for the simplest case when the energy applied to the system is fully absorbed. Similar calculations may be done for effective energies *u^eff^*, determined by Expression (87).

## 7. Translimiting States

The available information reports that the theory of translimiting states is still insufficiently developed [2]. Its elements will be set forth on the basis of solutions (72), (76) and (77).

**Figure 9:**
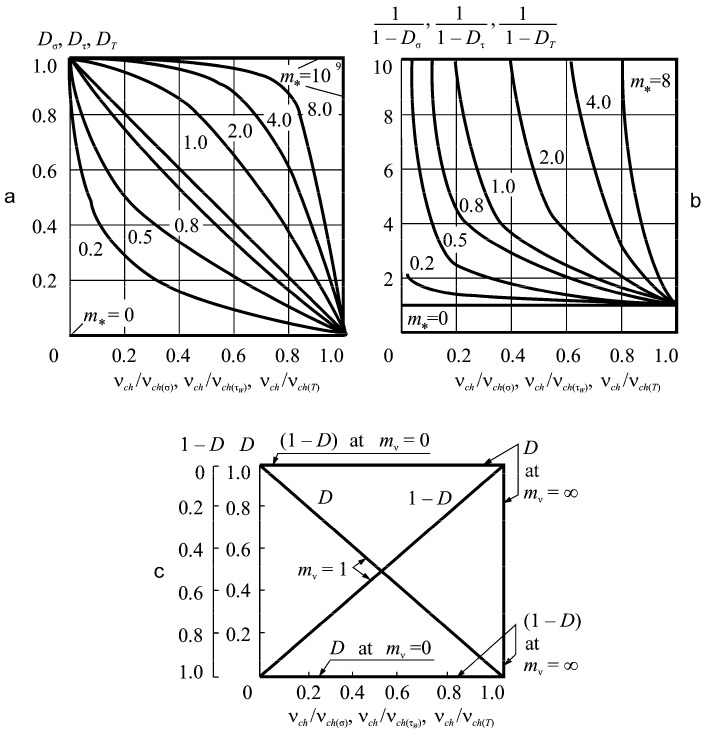
Effect of mechanical–chemical–thermal processes on the damage of a system (**a**) the dependence of parameters *D* on relative damage rate *v_ch_ */ *v_ch_*_(*)_; (**b**) influence of parameter *m_v_*; (**c**) specific cases analysis for *D* = 0,1.

Figure 9 analyzes the contribution of mechanical–chemical–thermal damage (parameters D) to reaching the limit state by the MTD system. Having analyzed Formulas (72), (76), and Figure 9, we concluded the following.

1. The growth of parameters *D* means that the relative damage speed *v_ch_*/*v_ch_*_(*)_ decreases (Figure 9a). Mechanical–chemical–thermal damage speeds up the process of reaching the limiting state by the MTD system. It is faster for the greater magnitude of *D* parameter and/or speed *v_ch_*_(*)_. 

2. Parameter *m_v_* greatly affects the system damage. The greater its effect, the larger this parameter is (Figure 9b). The MTD system is sensitive to mechanical load and temperature increase if electrochemical activity parameter *m_v_* > 5. In this case, the translimiting state may occur. For a state, the damageability measure (Equation (53)) becomes greater than unity ($\psi_{u}^{eff}$ > 1), while $\psi_{u}^{eff}$ = 1 in Equation (52) is enough to obtain the limiting state.

The first specific case in Figure 9c is *D* = 0. Electrochemical corrosion does not affect wear-fatigue damage. However electrochemical corrosion may happen. According to Formula (76), when *D* = 0 for *m_v_* = 1 we obtain:

$$1-\frac{v_{ch}}{v_{ch(*)}}b_{*}=0$$

Hence, the situation must be the following: b_*_ = 1 and *v_ch_* /*v_ch_*_(*)_ = 1. In this case, the corrosion speed is not influenced by mechanical or frictional stresses. So, there are threshold values of $\sigma^{0}$, $\tau_{w}^{0}$, and *T* ^0^ for a considered environment. The speed of corrosion for this environment according to Equation (77) stays the same at σ ≤ $\sigma^{0}$, $\tau_{w}\leq \tau_{w}^{0}$, and *T*_∑_ ≤ *T*^0^.

The second case is for *D* = 1, and hence, for 1/(1–*D*)→ ∞. Damage of explosive type happens in a system if $\psi_{u}^{eff}$→ ∞. In this case, it should be:

$$\frac{v_{ch}}{v_{ch(*)}}b_{*}=0$$

In case *v_ch_* = 0 is an impossible event, then it may be assumed that *v_ch_*_(*)_→∞. This is the condition for mechanical–chemical–thermal explosive event occurrence in a MTD system. This event is not just due to the environmental impact that is catastrophically increased by mechanical and temperature stress.

The damageability function of the MTD system (Equation (72)) can also be applied to the analysis of the system translimiting states. It can be done because of the possibility to take into account supercritical growth of frictional, mechanical, thermodynamic, and electrochemical loading by Equations (73)–(76), i.e.,

(137) $$1\leq \psi_{u}^{eff}=\Lambda_{T\backslash M}\left[\psi_{T(ch)}+\Lambda_{n\backslash \tau }(\psi_{n(ch)}+\psi_{\tau (ch)})\right]\leq \infty$$

According to Equation (137), many translimiting states could be described by the $\psi_{u}^{eff}>1$ condition. It may happen if the system limiting by damageability state occurs not only at one but at many points (elementary volumes) that constitute a dangerous volume. It could be assumed that there must exist many different types of such states. 

Although the above criterion Equations (43), (47), (52), (58), (72) and (77) are constructed for the analysis of energy limiting state conditions, they could also be applied to the description of different translimiting states under supercritical loads (at fires, disasters, accidents, explosions, etc.).

A different general way to analyze the translimiting states uses a damage space defined by volume damageability measures according to Equations (59) and (64):

(138) $$0\leq \omega_{ij}=\frac{V_{ij}}{V_{0}}\leq 1$$

On the basis of Equations (72)–(76), volume (space) damageability measures can be defined as:

(139) $$\begin{array}{c} \omega_{\sigma (ch)}=\frac{V_{P\gamma }}{V_{0}(1-D_{\sigma })}\\ \omega_{\tau (ch)}=\frac{S_{P\gamma }}{S_{0}(1-D_{\tau })}\\ \omega_{T(ch)}=\frac{V_{T\gamma }}{V_{0}(1-D_{T})} \end{array}$$

where, $V_{0}$, $S_{k}$ are the working volumes. Criterion (77) can then be written with regard to (139):

(140) $$\Lambda_{T\backslash M}\left[\frac{V_{T\gamma }}{V_{0}(1-D_{T})}+\Lambda_{\sigma \backslash \tau }\left(\frac{V_{P\gamma }}{V_{0}(1-D_{\sigma })}+\frac{S_{P\gamma }}{S_{0}(1-D_{\tau })}\right)\right]=1$$

The advantage of Equation (140) is the following. Here, the interaction of dangerous volumes [2,4] at different loads when forming the limiting state of MTD systems is taken into account. Also, dangerous volumes are influenced by different metallurgical, technological, and structural factors as it is shown in Equation (59). 

If interatomic bond ruptures are analyzed only at a dangerous section of a body at all its points (elementary surfaces) $\left(u_{\Sigma }^{eff}=u_{0}\right)$, then it divides into two parts corresponding to $\omega_{\Sigma }$= 1, but if loads (mechanical, electrochemical, thermodynamic, etc.) are combined in such a way that “all” interatomic bonds undergo rupture over this section, then there occurs the process called the object disintegration. It corresponds to $\omega_{\Sigma }^{*}=\infty$:

(141) $$1\leq \omega_{\Sigma }^{*}=\Lambda_{T\backslash M}\left[\left(\omega_{\sigma (ch)}+\omega_{\tau (ch)}\right)\Lambda_{\sigma \backslash \tau }+\omega_{T(ch)}\right]\leq \infty$$

Naturally, Equation (141) is similar to (137). Their difference lies in the fact that condition (137) is formulated as energy damageability measures while condition (141) is formulated as volume (space) damageability measures.

Table 1 contains a classification of object states by volume damageability. 

Irreversible damageability events in the MTD system can be interpreted using the failure probability.

**Figure 10:**
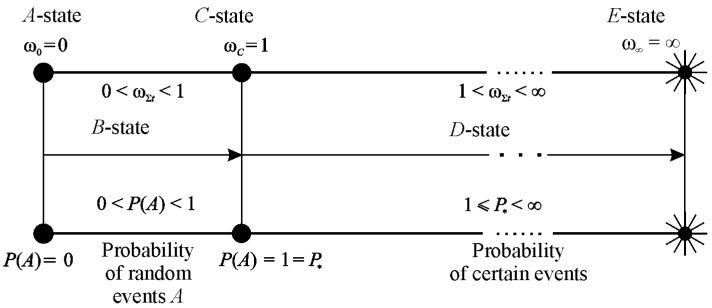
Connection between system damageability and event probability.

If

(142) $$0\leq P(\omega_{\Sigma })\leq 1$$

is the traditional probability of failure by damageability ($0\leq \psi_{\Sigma }^{}\leq 1$) within the time interval ($t_{0},\;T_{⊕}$) (item XIV), then $P(\omega_{\Sigma }=\omega_{c}=1)=1$ is the reliable probability of unconditional functional failure. In case of supercritical states, the concept of reliable probabilities [79] can be formulated (see Figure 10):

(143) $$1<P_{*}\left(\omega_{\Sigma }^{*}\right)\leq \infty$$

These supercritical damages $1<\omega_{\Sigma }^{*}<\infty$ are consistent with numerous and innumerable shapes and sizes of particles forming during the system degradation (disintegration). 

Data in Table 1 can be interpreted in the following way. If

(144) $$\omega_{\Sigma }^{*}\to \infty .$$

then forming particles should have absolute size, according to Equation (32):

(145) $$d_{\omega }^{*}\to 0.$$

To a first approximation, we assume a logarithmic relationship between $d_{\omega }$ and $\omega_{\sum }$. Then,

(146) $$d_{\omega }^{*}=e^{-\omega_{\Sigma }^{*}}\;\mathrm{or}\;\omega_{\Sigma }^{*}=-\ln d_{\omega }^{*}$$

**Figure 11:**
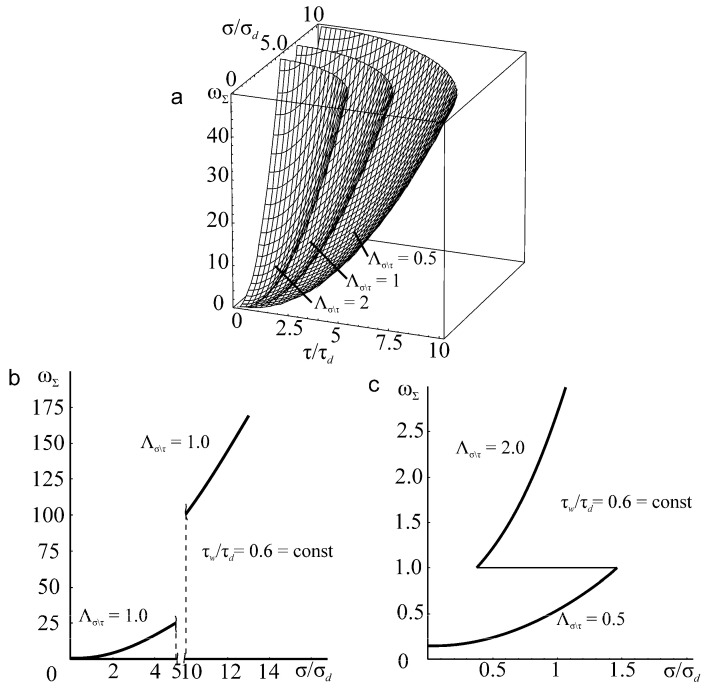
The surfaces of (**a**) damageability, (**b**) ω_Σ_ and (**c**) determined by parameters τ/τ***_d_*** > 0, σ/σ*_d_* > 0, Λ_σ\__τ_ > 0.

As follows from the foregoing, all MTD system states (see Figure 11) caused by both continuous and discontinuous change of governing parameters are predicted by corresponding Equations (137) and/or (141). The law of MTD system decomposition (decay) can be formulated the in the following way:

(147) $$\sum m_{V_{ijT}}=m_{V_{0}}.$$

Law (147) implies the conservation of mass of the system regardless of the conditions of its degradation and disintegration. The mass of disintegrated parts (particles) $\sum m_{V_{ijT}}$ (independently of their size) cannot exceed the initial system mass $m_{V_{0}}$.

## 8. Analysis and Generalization of Experimental Data

It is extremely difficult to experimentally verify generalized criterion (72) of the MTD system limiting state due to the lack of experimental data. Below, we consider some particular cases of criterion (77) in the form of (78).

Let us obtain some applied formulas basing on criterion (78). Conditions of purely thermal at σ = 0 and τ*_w_* = 0 or purely mechanical damage at *T*_Σ_→0 are the following:

(148) $$a_{T}T_{\Sigma }=u_{0};$$

(149) $$\Lambda_{n\text{\textbackslash{}}\tau }\left(a_{n}\sigma^{2}+a_{\tau }\tau_{w}^{2}\right)=u_{0}.$$

Isothermal mechanical fatigue at τ_w_ = 0 could be described by:

(150) $$\Lambda_{M\backslash T}\left(a_{T}T_{\Sigma }+a_{n}\sigma^{2}\right)=u_{0},$$

and isothermal frictional fatigue at σ = 0:

(151) $$\Lambda_{M\backslash T}\left(a_{T}T_{\Sigma }+a_{\tau }\tau_{w}^{2}\right)=u_{0}.$$

The analysis of these specific criteria drives us to the following conclusions.

(1) Increase of load parameters (σ, τ_w_, *T*_Σ_, *D*) yields the corresponding acceleration of reaching the limiting state (*u*_0_).

(2) System limiting state can also be reached by increasing only one (any) of the load parameters (when the values of other parameters are invariable). 

(3) If Λ > 1, the system damageability increases (i.e., the processes of its softening are dominant). If Λ < 1, damageability decreases (i.e., the processes of system hardening appear are dominant) in comparison to the damageability due to only a collective action of load parameters (when the dialectic interaction of irreversible damages is not allowed for).

The last conclusion also results from a fundamentally new approach to constructing the criterion of the limiting state of MTD systems [80]. According to this approach, not the mutual influence of the factors, but the interaction (Λ ⋛ 1) of phenomena, is responsible for damageability processes in the MTD system [1,45,46,47,48,49,50,51,52,80]. In this regard, we synthesized the results of more than 600 diverse experimental data. This permitted the generalized MTD function of critical damageability states to be revealed.

We turn to a special case of criterion (78)—isothermal mechanical fatigue. From Equation (150) we have:

(152) $$\log\sigma_{-1T}=\frac{1}{2}\log C_{T};\;C_{T}=\left[u_{0}/\Lambda_{M\backslash T}-a_{T}T_{\Sigma }\right]\cdot \frac{1}{a_{n}}$$

**Figure 12:**
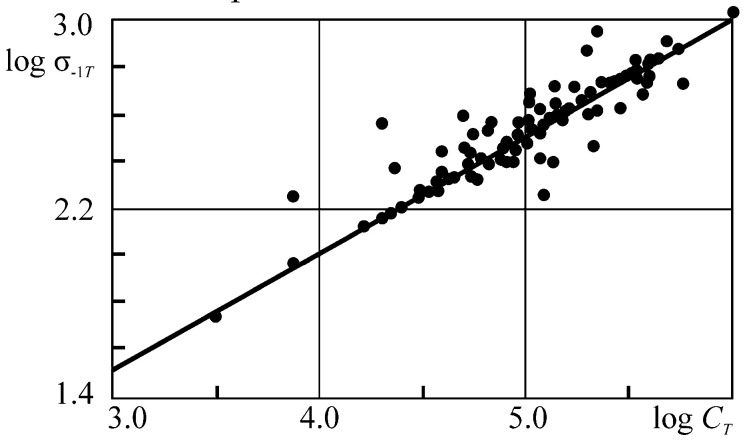
Fatigue limits of structural steels versus parameter of thermomechanical resistance *C_T_*.

Figure 12 convincingly confirms the dependence (Equation (152)) of σ_−1*T*_ on the parameter of thermomechanical resistance *C_T_* for numerous steels of different grade tested for fatigue at different conditions [78,81,82]. The *C_T_* magnitude changes by a factor of 100 or more and the value of fatigue limit $\sigma_{-1T}$ by a factor of 10 or more. Testing temperature was thus varied from the helium temperature to 0.8 *T_s_* (*T_s_* is the melting point). As shown in Figure 12, Equation (152) adequately describes the results of more than 150 experiments.

**Figure 13:**
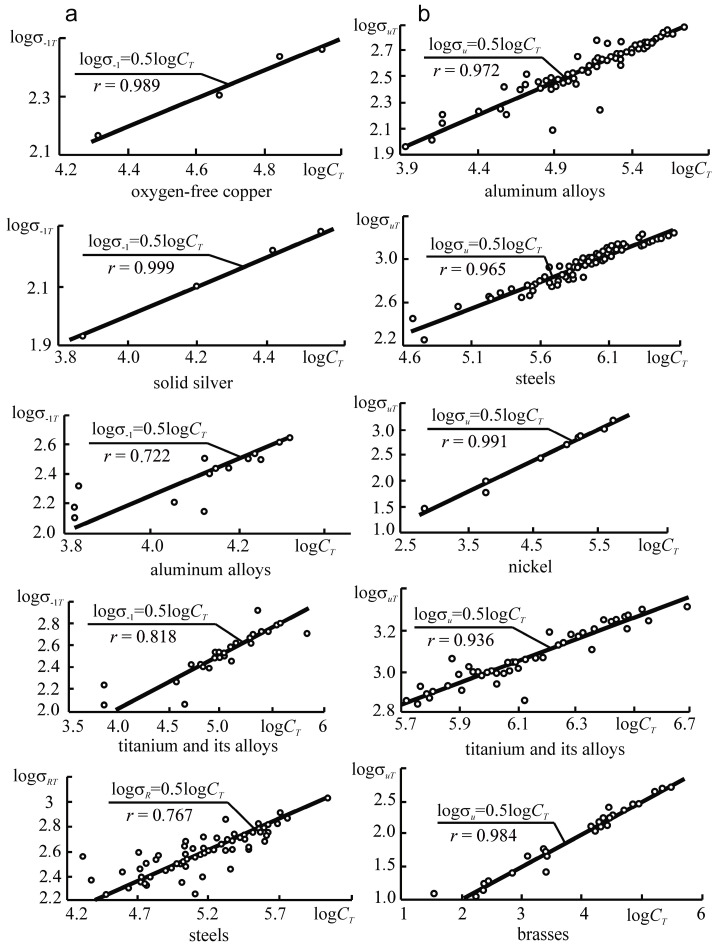
Dependences (**a**) σ_–1_ (*C_T_*) and (**b**) σ*_u_* (*C_T_*) for different metals.

Equation (152) was also checked for different metals according to the results of fatigue test carried out by different authors (Figure 13a). In References [78,82], it is possible to find the list of references.

Figure 13b*,* analyzes the results of tensile tests under different temperatures (σ*_uT_*—the strength limit). In Equation (152), σ_–1_ = σ*_uT_*. The correlation coefficient is obviously very high even for the rare cases: *r* = 0.722. In most cases, the coefficient exceeds *r* = 0.9 for more than 300 test results that were analyzed. References [78,82] also contain other examples of successful experimental verification of criterion (152). We can hope that even more general criteria given by Equations (77) and (78) will be acceptable in applications.

**Figure 14:**
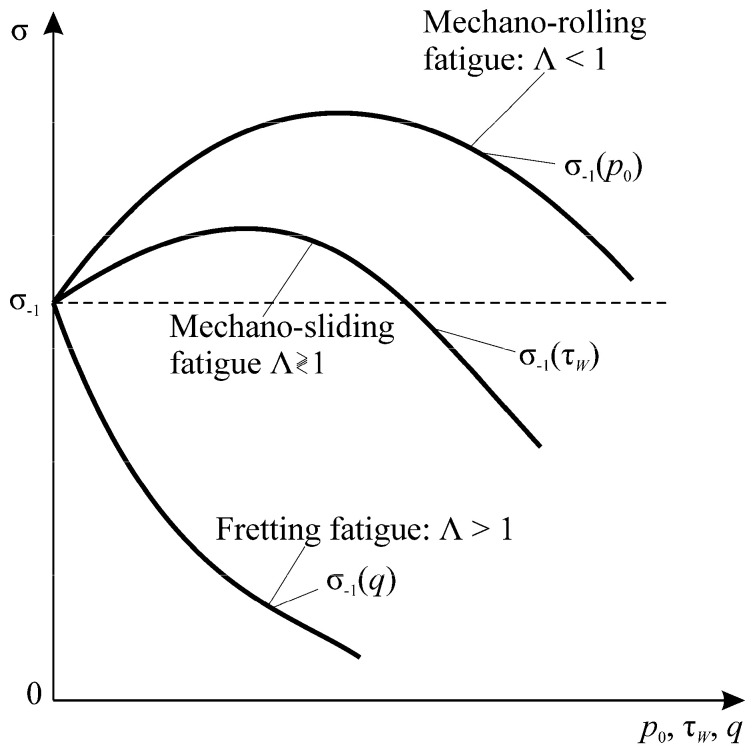
Main features of Λ-interactions in the tribo-fatigue system.

As said above, criterion Equation (149) is valid for $\sigma \leq \sigma_{u}$. For specific testing, the conditions $\tau_{W}$ can be treated as the largest contact pressure ($p_{0}$) at the contact zone center under rolling. It can also be treated as the sliding stress $\tau_{w}$ or as the nominal (average) pressure *p_a_* at the contact area under sliding, or as the pressure (*q*) at fretting. If $\sigma =\sigma_{-1}$ is fixed, where $\sigma_{-1}<<\sigma_{u}$, then Equation (28) can be presented in the form of the diagram of the limiting states of tribo-fatigue systems [2,81,82] (Figure 14). 

Criterion Equation (149) clearly distinguishes the zones of realization of spontaneous hardening–softening processes (interaction function Λ ⋛ 1). Figure 14 yields the above obvious conclusions: if Λ < 1, then the self-hardening system (during tests or during operation at these conditions) is considered. If Λ > 1, then the system turns to be self-softening. If Λ < 1 is found to convert into Λ > 1, then it implies that because of changing the determining operation conditions, hardening processes are replaced by softening ones.

**Figure 15:**
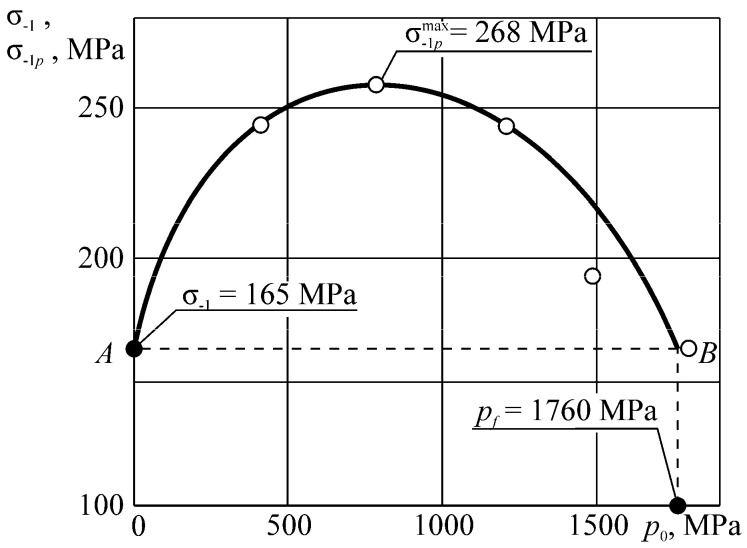
Influence of rolling friction on the resistance to mechano-rolling fatigue in the tests of the tribo-fatigue steel 45 (shaft)/steel 25 HGT (roller) system.

**Figure 16:**
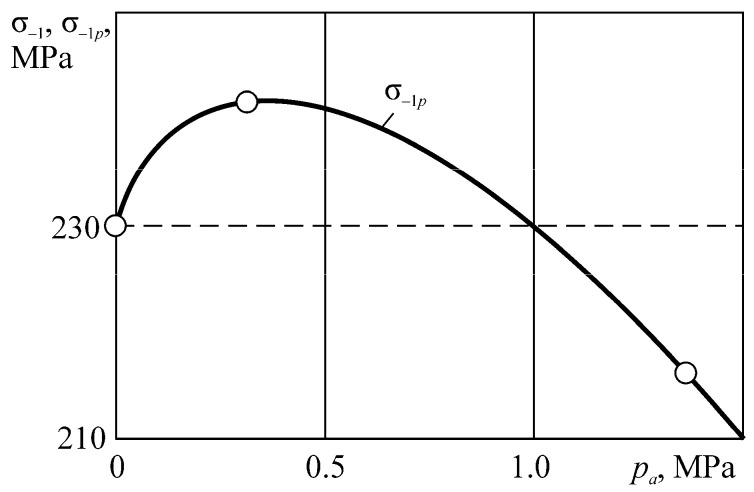
Limiting stresses versus the contact pressure for the tribo-fatigue steel 45 (shaft)/cast iron (partial bearing insert) system.

**Figure 17:**
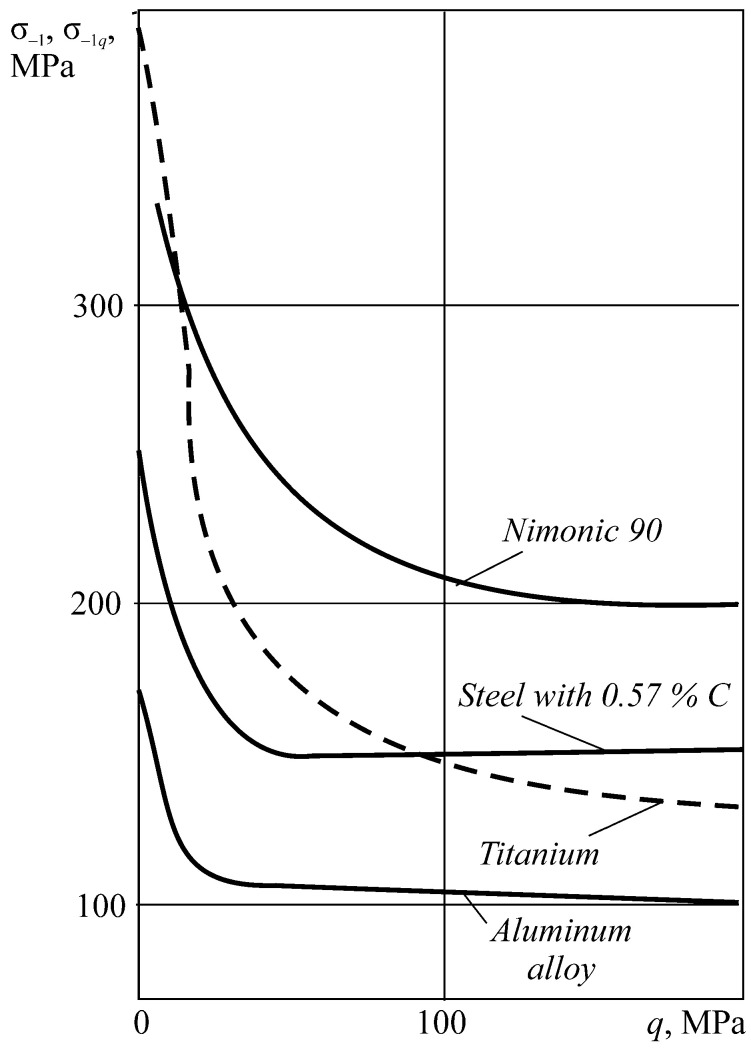
Contact pressure versus the fatigue limit under fretting fatigue.

Figure 15, Figure 16, Figure 17, illustrate the additional experimental verification of these conditions. Note that for spontaneous hardening (for Λ < 1, Figure 14, Figure 15, Figure 16), the stress limit in wear-fatigue tests is higher than in routine fatigue tests. In these conditions, the friction and wear processes become "useful". Numerous works (see Reference [83]) illustrate that dosed wear in real tribo-fatigue systems (wheel/rail) causes an appropriate growth of their fatigue strength. When Λ >> 1 (Figure 14), they lead to a strong damageability growth: the fatigue limit decreases with increasing contact pressure *q* by a factor of 2…3. In addition, there are many works (see Reference [84]), showing that the system wear suddenly decreases the fatigue strength.

Table 2 and Table 3 summarize different physical signs (often encountered in practice) of the limiting state that can find use in relevant research areas.

As for the determination of the parameters Λ_*M\T*_ and Λ_*n\τ*_, References [2,78] show that the parameter Λ_*n\τ*_ is the function of the relative skewness coefficient of wear-fatigue damage:

(153) $${\bar{\rho }}_{n\backslash \tau }={\left(\frac{\tau_{w}}{\tau_{f}}\right)}^{2}{\left(\frac{\sigma_{-1}}{\sigma }\right)}^{2}$$

**Figure 18:**
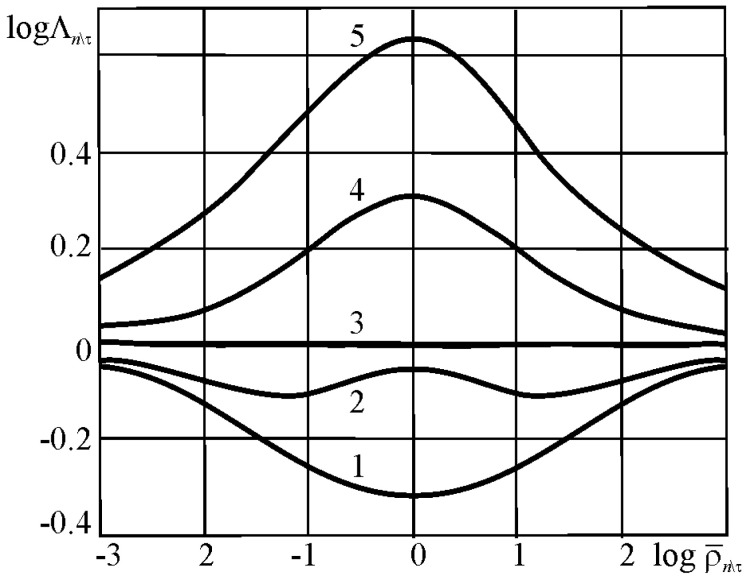
Typical plots of the character and direction of hardening–softening processes (Λ ⋛ 1) versus the skewness coefficient of the damageability processes $\bar{\rho }$: 1, 2–mechano-rolling fatigue; 2, 3, 4–mechano-sliding fatigue; 4, 5–fretting-fatigue.

Hence ${\bar{\rho }}_{n\backslash \tau }$ depends not only on absolute values of effective ($\sigma ,\;\tau_{w}$) and limiting ($\sigma_{-1},\;\tau_{f}$) stresses, but also on their ratios: $\tau_{w}/\sigma$, $\sigma_{-1}\;/\;\tau_{f}$, $\sigma_{-1}\;/\sigma$, $\tau_{w}/\tau_{f}$⋛ 1. This means that very different patterns of accumulation of irreversible damages occur depending on the realization of inequalities σ ⋛ $\sigma_{-1}$, $\tau_{w}$⋛ $\tau_{f}$. This conclusion is supported by the known experimental results and theoretical models. Figure 18 depicts the analysis of the possible dependences $\log\Lambda_{n\backslash \tau }-\log{\bar{\rho }}_{n\backslash \tau }$ based on References [2,78]. A more detailed analysis of the interdependences $\Lambda_{n\backslash \tau }\left({\bar{\rho }}_{n\backslash \tau }\right)$ can be found in References [2,78].

Here, σ_lim_ is the limiting stress, *T_s_* is the melting point, *t*_lim_ is the longevity, σ*_ij_* is the stress (strain) tensors, *T_Σ_* is the temperature due to all heat sources, σ*_ijT_* is the stress tensor in the isothermal (*T_Σ_* = const) state, σ*_ijT_* and *T_Σ_* are the stress-strain state and the thermodynamic state, respectively, and σ*_ijT_*, *T_Σ_,* and *t* are the stress-strain state and the thermodynamic state in time, respectively

**Figure 19:**
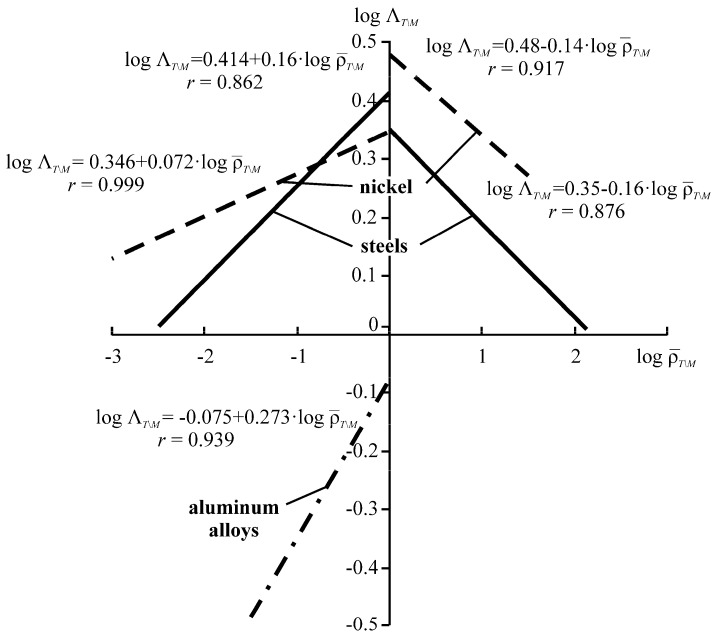
Logarithmic plots of $\Lambda_{T\backslash M}({\bar{\rho }}_{T\backslash M})$ built on the basis of the experimental data.

The plot of the $\Lambda_{T\backslash M}$ interactions versus the parameter ${\bar{\rho }}_{T\backslash M}$ can be analyzed in a similar way. Figure 19 illustrates the plots for steel, aluminum alloys, and nickel in the double logarithmic coordinates (according to the extensive experimental results [2,78]). The correlation coefficient *r* appears to be very high from 0.862 to 0.999. The plot of $\Lambda_{T\backslash M}({\bar{\rho }}_{T\backslash M})$ suddenly changes for lg ${\bar{\rho }}_{T\backslash M}$ = 0 (${\bar{\rho }}_{T\backslash M}$=1) when thermal and stress damages turn to be in equilibrium (in comparison to the similar changes in the dependencies in Figure 18).

For steels and nickel at ${\bar{\rho }}_{T\backslash M}$ < 1, the direct dependence is found between $\Lambda_{T\backslash M}$ and ${\bar{\rho }}_{T\backslash M}$, and at ${\bar{\rho }}_{T\backslash M}$ > 1 it becomes inverse. For aluminum alloys, the dependence $\Lambda_{T\backslash M}$ ^(^${\bar{\rho }}_{T\backslash M}$^)^ is also direct, but located (at ${\bar{\rho }}_{T\backslash M}$ < 1) in the III quadrant.

It is experimentally confirmed that the interaction parameter $\Lambda_{T\backslash M}$ is sensitive not only to the effective thermal-to-mechanical energy ratio, but also to the structure and composition (or nature) of metal materials. The last conclusion is also valid for the parameter $\Lambda_{n\backslash \tau }$: its numerical values appear to be significantly different, for example, for metal/metal and metal/polymer active systems even in the case when the ratios ${\sigma \text{\textbackslash{}}\sigma }_{-1}$ and $\tau_{w}\backslash \tau_{f}$ are identical for them.

In this section we briefly analyze the data of more than 600 tests of metals and their alloys (at isothermal conditions) obtained by many authors.

**Figure 20:**
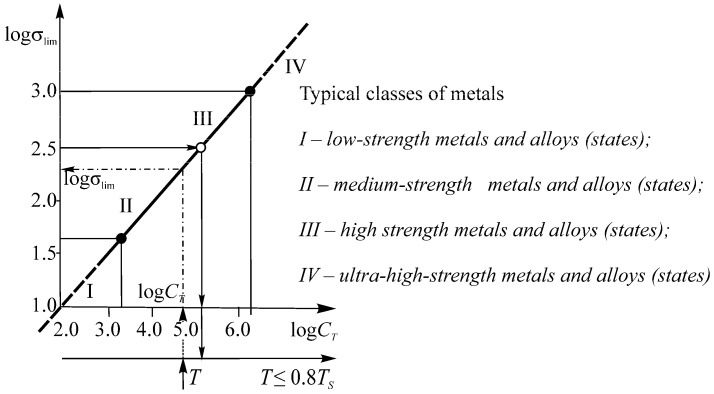
Generalized MTD function of the limiting states of metals and alloys ($\sigma_{\lim}\leq \sigma_{u};\;T\leq 0,8T_{S}$), model (7).

It was found that the thermodynamic dependence of limiting stresses can be presented in the logσ_lim_ − log*C_T_* coordinates (Figure 12 and Figure 13 and Formula (152)), where the function

(154) $$C_{T}=C_{T}\left(T,\;u_{0},\;a_{n},\;a_{T},\;\Lambda_{M\backslash T}\right)$$

is satisfactory at static tension (σ_lim_ = σ*_u_*) and fatigue fracture (σ_lim_ = σ_–1_) for numerous and various metal materials (steels; aluminum, titanium, alloys, etc.). In addition, interrelation (152) appears to be valid practically within the entire possible interval of temperature ($T\leq 0,8T_{S}$) and stress (σ ≤ σ*_u_*) varying with the correlation coefficient *r* = 0.7 in the specific cases and usually with *r* > 0.9. Model (152) then turns to be fundamental (Figure 20). The simplified model may seem dubious because in the known works (see Reference [85,86]), the explicit temperature dependence of limiting stresses is described by complex curves. This is attributed to the changes in the failure mechanisms of various materials at different testing conditions: normal, operating, and other temperatures.

Nevertheless, the fundamental nature of model (152) is supported experimentally (Figure 12 and Figure 13).

From the theoretical standpoint, we can say the following in favor of model (152). It has four parameters (Formula (154)), one of them (*u*_0_) is a fundamental constant of substance (Formulas (48) and (49) in Reference [80]), and the other two (*a_T_*, *a_n_*) are defined by the boundary conditions as the relations *u*_0_ and physical constants σ*_d_* and *T_d_* of a given material [78]:

(155) $$a_{\sigma }=u_{0}/\;\sigma_{d}^{2},\;a_{T}=u_{0}/\sigma_{d}.$$

The methods to determine σ*_d_* and *T_d_* are outlined in References [2,78]. Here, we remind that material failure limit σ*_d_* is obtained at tension fof *T_Σ_* → 0. Failure temperature *T_d_* is obtained at the body heating for σ=0 . Therefore, in a general case, the accumulation of damages and failure due to mechanical stresses and thermal activation of these stresses in time is taken into account [67]. Finally, as it was briefly discussed above and given in References [4,76], the function 1 Λ*_M\T_* ⋛ 1 takes into account damage interaction considering the change of ratio of σ ⋛ σ*_lim_*. Known studies [2,4,86] repeatedly and convincingly prove that this ratio determines the mechanism and character damage at different types of strain. The role of thermal fluctuations (*T_Σ_* < *T_d_*) is also studied in detail in References [67,68].

**Figure 21:**
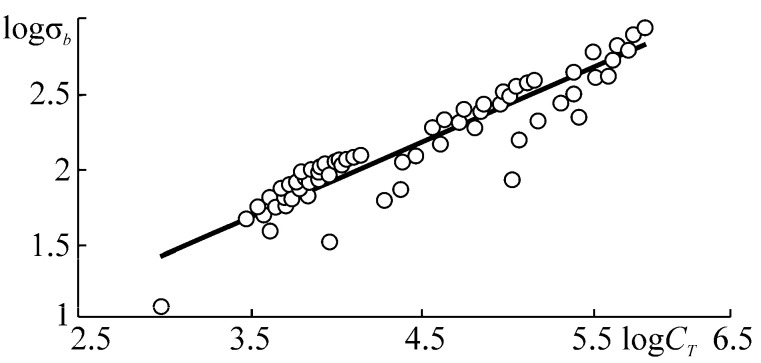
Dependence $\sigma_{u}\left(C_{T}\right)$ for polymer materials.

Further analysis of non-metalic (polymer) materials proves the fundamental nature of model (152). Table 4 and Figure 21 contain the analysis results of the polymer tests based on the experimental data [87]. It is obvious that model (152) is confirmed with the correlation coefficient *r* = 0.917. It should be noted that these test results are obtained not only for usual specimens (of ~5 mm diameter). Also, the results of tests of thin polymer films and threads are used not only under tensile deformation but also under torsion and bending. Large deviation of some points from the basic straight line could be explained by conventional accepting $\Lambda_{M\backslash T}\;$= 1 because of the lack of test data in order to estimate its actual value.

**Figure 22:**
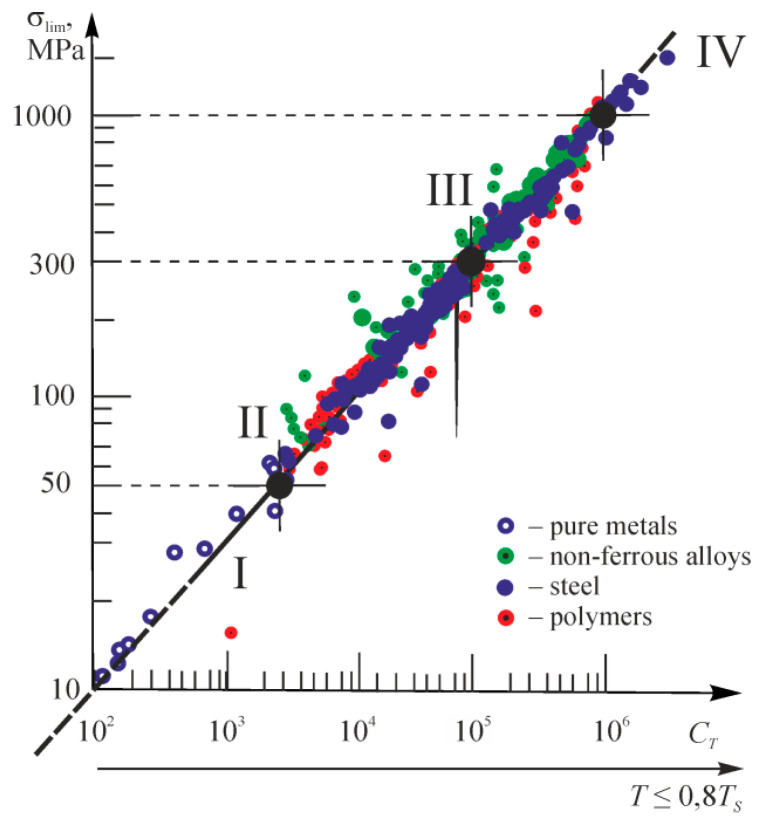
Experimentally verified MTD function of the critical by damageability states of metal and polymer materials.

Figure 22 illustrates the generalized experimentally verified MTD function of the limiting (by damageability) states. Figure 12 and Figure 13 (compared to Figure 22) depict relatively large deviations of particular experimental points from the predicted ones. There are two reasons for that. The first one is that available references may have no data for a correct assessment of required parameters. The second reason is that the conducted experiments reveal significant errors, or they were not methodically correct.

Note that model (152) may seem to be non-fundamental because of its simplicity. However, we remind the classic dictum: the fundamental dependence cannot be complicated (or: every law is described by the simplest formula).

Model (152) can then serve for prediction of mechanical behavior of materials in the thermodynamic medium (shown by the arrows from T to $\sigma_{\lim}$ in Figure 20):

(156) $$T\overset{\overset{\underline{u_{0},\;\Lambda_{M\backslash T}}}{↓}}{\underset{\underset{\bar{a_{n},\;a_{T}}}{↑}}{\to }}\log C_{T}\to \log\sigma_{\lim}\left(T,u_{0},a_{n},a_{T},\Lambda_{M\backslash T}\right)\to \sigma_{\lim(T)}.$$

The parameters T, $a_{T}$, and $\Lambda_{M\backslash T}\;$ are responsible for the medium state in Equation (153).

Predictions by Equations (152) and (156) could be applied to the materials of different nature and structure. They are irrespective of damage and fracture mechanisms under static and cyclic loads. 

Of course, because of the linearity of Equation (152), the reverse prediction could be possible and effective. In case a mechanical state of material (defined by the parameters $u_{0}$, $\sigma_{\lim(T)}$) is known, then the requirements can be formulated to the medium (defined by the parameters T, $a_{T}$, $\Lambda_{M\backslash T}\;$) where the system can work (the arrows from σ_lim_ to *T* in Figure 20):

(157) $$\sigma_{\lim(T)}\to \log\sigma_{\lim(T)}\underset{\underset{\bar{a_{n},\;a_{T}}}{↑}}{\overset{\overset{\underline{u_{0},\;\Lambda_{M\backslash T}}}{↓}}{\to }}\log C_{T}\to C_{T}\left(T,u_{0},a_{n},a_{\tau },\Lambda_{M\backslash T}\right)\to T.$$

**Figure 23:**
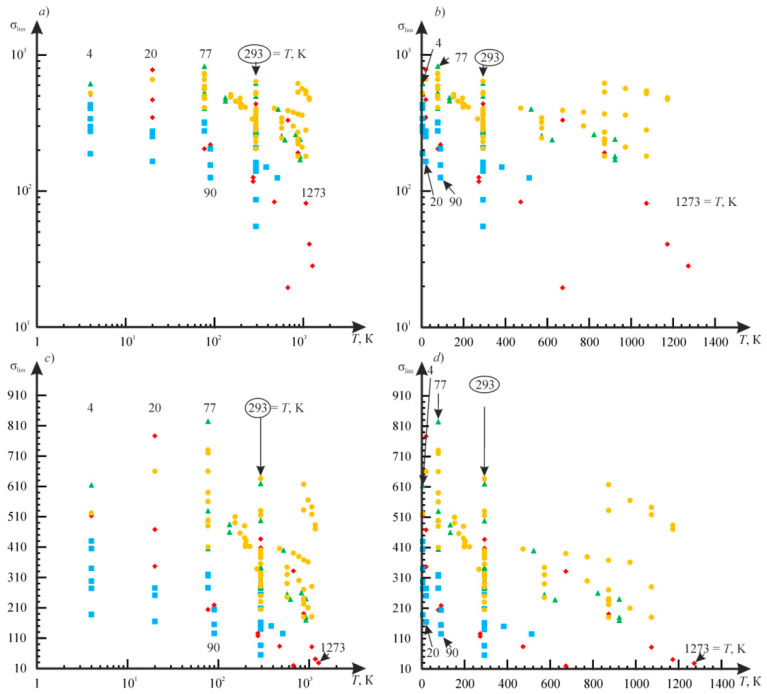
Dependencies of metals fatigue limit (according to 136 results of tests of many authors) on temperature.

Note that the attempts to construct an explicit temperature dependence of limiting stresses in uniform, semi-logarithmic, and logarithmic coordinates for various materials and different testing conditions are quite ineffective (Figure 23). We will further briefly analyze a more complex problem of the MTD system operation in the medium under the processes of thermal corrosion and corrosion at stress. From Equation (77), at $\tau_{w}=0$ we have

(158) $$\Lambda_{M\backslash T}\left(\frac{a_{T}}{1-D_{T}}T_{\Sigma }+\frac{a_{n}}{1-D_{n}}\sigma^{2}\right)=u_{0}$$

Upon simple manipulations we obtain:

(159) $$\sigma_{\lim(T,\;ch)}=\frac{1}{2}\log C_{T(ch)}$$

where the parameter of thermal resistance to corrosion at stress is:

(160) $$C_{T(ch)}=C_{T(ch)}\left(T,\;u_{0},\;a_{n},\;a_{T},\;\Lambda_{M\backslash T},\;v_{ch},\;v_{ch(\sigma )},\;m_{v(\sigma )},\;v_{ch(T)},\;m_{v(T)}\right).$$

It is seen that models (152) and (159) are fundamentally (and formally) identical. They differ because corresponding functions (154) and (160) use the parameters describing the damageability processes characteristic of the analyzed phenomena. In function (160), parameters $v_{ch},$ $v_{ch(\sigma )},$ $m_{v(\sigma )},$ $v_{ch(T)},$ $m_{v(T)}$ describe the processes of thermal corrosion at stress (Formula (76) in Reference [80]). Based on models (159) and (160), it is easy to develop prediction algorithms (type (156) and (157)) of resistance to thermal corrosion at stress. 

A detailed analysis of models (157) and (160) is beyond the scope of the present work. It can be made in the future as applications to the novel results described in References [3,4,76].

It should be noted that solutions (77)–(151) can be analyzed in a similar way for other operating (or testing) conditions.

## 9. Discussion

The foregoing gives three main conclusions:

1. Damage is a fundamental physical property (and a functional duty) of any system and all its elements. 

2. Damageability of each object (any existing one) inevitably grows up to its death–decomposition (disintegration) into a set of particles of arbitrarily small size, i.e., it is the unidirectional time process.

3. Evolution of the system by damageability is characteristic not only of the unity and struggle of opposites, but also of the directivity of various and complex physical hardening–softening processes (depending on the load and time level). It means that the Λ-function of interaction of different-nature damages can take three classes of values: (1) Λ < 1 when the hardening process is dominant, (2) Λ > 1 when the softening process is dominant, and (3) Λ = 1 when a stable hardening-to-softening process ratio is found.

So, the first law of mechanothermodynamics states that the evolution of any system inevitably needs a unidirectional process of its damage and disintegration, finally, into an infinitely large number of small components (fragments, atoms, elementary particles, etc.). In fact, it is equivalent to the recognition of the evolution endlessness, if it is taken into account that disintegration products of any system become a construction material for new systems. Thus, the evolution hysteresis is formed.

The second law of mechanothermodynamics states: interaction Λ-functions must take three classes of values (Λ ⋛ 1) to describe not only the unity and struggle of opposites, but also the directivity of physical hardening–softening processes in the system, i.e., the system evolution by damageability [50,52,76].

**Figure 24:**
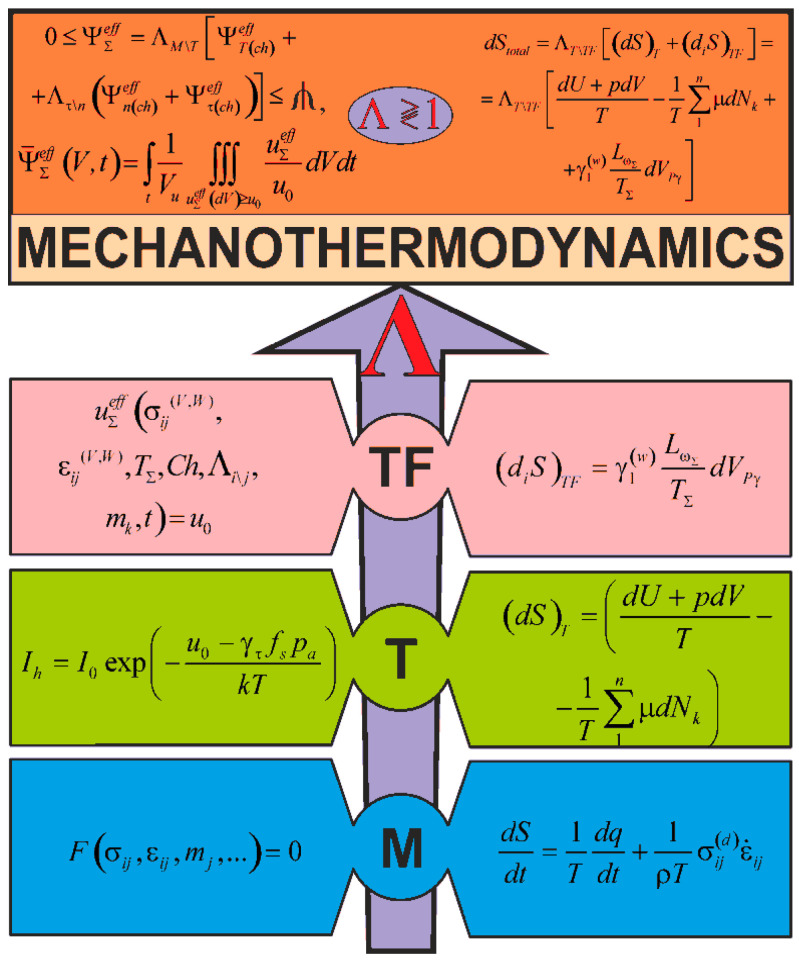
Energy (**left**) and entropy (**right**) approaches to developing mechanothermodynamics (M: mechanics, T: thermodynamics, TF: tribo-fatigue).

Figure 24 generalizes the above results [1,2]. It is seen that the system state can be equivalently described in terms of either energy or entropy. The main drawback of such descriptions is the known unreality of energy, and hence, of entropy: physical energy carriers are not detected and, apparently, do not exist. As Feynman [88] said, figuratively, they cannot be touched. Damages are completely different: they are physically real, can be touched, and actually define any of the conceivable states of material bodies and systems. The kinetic process of their accumulation, as well as the time stream, is inevitable and unidirectional. 

**Figure 25:**
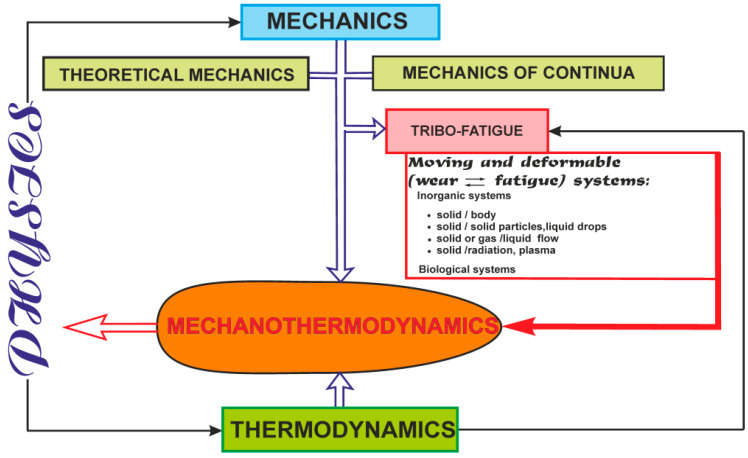
Ways towards mechanothermodynamics as a new branch of physics.

An attempt was made to formulate the basic principles of a new (or, better to say, integrated) physical discipline–mechanothermodynamics with the use of the energy principles. This discipline combines two branches of physics in order not to argue or not to compete with each other, but to take a fresh look at the MTD system evolution (Figure 25).

**Figure 26:**
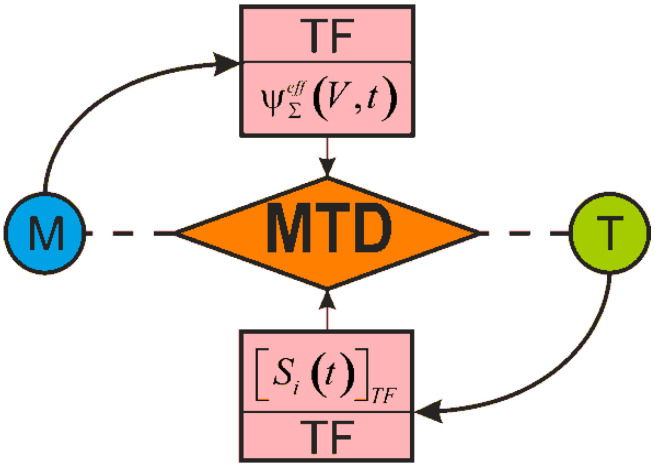
Tribo-fatigue bridges from mechanics (**M**) and thermodynamics (**T**) to mechanothermodynamics (MTD) are denoted by the solid lines with arrows and the unrealized ways (during more than 150 years, from M or T to MTD)—by the dashed lines.

Figure 26 shows that the principles of mechanothermodynamics can be formulated in two ways: (1) mechanics → tribo-fatigue → mechanothermodynamics and (2) thermodynamics → tribo-fatigue → mechanothermodynamics. So, tribo-fatigue has become a bridge to pass from mechanics and thermodynamics to mechanothermodynamics.

The fact that both ways lead to one objective and, finally, yield the same (unified) result, means that the above-mentioned two methodologies of analysis are valid, correct, and do not contradict each other.

## 10. Conclusions

1. It is shown that a generalized physical discipline—mechanothermodynamics—can be created by making two main bridges. The first one is the tribo-fatigue entropy that allowed transfer from thermodynamics to mechanics. The second one is the fundamental tribo-fatigue understanding of irreversibility of damage of everything that allowed transfer from mechanics to thermodynamics. 

2. Main principles (I–XV) founding the general theory of evolution of MTD systems were formulated. 

The following models and theories were developed:

- Limiting state energy theory (Section 3).
- Damageability energy theory (Section 4).
- Fundamentals of electrochemical damageability theory (Section 5).
- Elements of MTD system transmitting state theory (Section 6).

3. Procedures and methods of calculation of effective (dangerous) energy expended for generation, accumulation, and motion of irreversible damages were developed (Formulas (79)–(83) and the text related to them).

4. Fundamentals of the theory of Λ-interaction between damages due to different loads of nature: thermodynamic, mechanical, etc. (Formulas (69), (70) and (155) and the text related to them) were outlined. This theory allows consideration of the effect of accidental hardening–softening processes on the limiting by damageability state of MTD.

5. The relationship between the damages of the system and the event probability (Figure 10) in the course of its evolution was analyzed. The idea of reliable damageability probability 1 <P_*_ <∞ at the stage of translimiting states was proposed.

6. The physical signs and specific characteristics of limiting states of objects and systems (Table 2 and Table 3) were given. These may be of use for specialists in the relevant research areas.

7. Practically, a unified MTD function of critical by damageability (limiting) states of polymer and metal materials working under different and complex conditions (Formula (152) and Figure 15) was obtained in the present work. The analysis of more than 600 experimental results (Figure 7, Figure 8, Figure 14, Figure 16, Figure 17) showed the fundamental nature of this function since it is applicable for high-, average-, and low-strength states of alloys, pure metals, and polymers. MTD function can be used for a wide range of medium temperatures (from 0.8 *T_S_* where *T_S_* is the melting point of material to temperature of helium), limiting values of mechanical stresses (up to the limit of strength under static loading), and the fatigue life of 10^6^…10^8^ cycles. This function can effectively predict the behavior of specific MTD systems at different working (testing) conditions (procedures (156) and (157)). Models (159) and (160) were proposed to describe the effects of corrosion at stress and thermal corrosion on the change in materials’ limiting states.

In conclusion, it should be noted that the research in mechanothermodynamics is in its initial stage. Expanding and deepening the front of research in this promising new area of knowledge could be expected in the near future. 

## Figures and Tables

**Table 1:** Characteristics of the states of objects.

***A*-state**	**Undamaged**	$\omega_{\Sigma }$ **= 0**	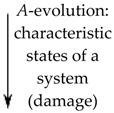
*B*-state	Damaged	0 < $\omega_{\Sigma }$ < 1	
*C*-state	Critical (limiting)	$\omega_{\Sigma }$ =1 = $\omega_{c}$	
*D*-state	Supercritical (translimiting)	1 < $\omega_{\Sigma }^{*}<\infty$	
*E*-state	Disintegration	$\omega_{\Sigma }^{*}=\infty$	

**Table 2:** Main signs of the physical state.

Energy State		Condition of Reaching the Limiting (Critical) State
Symbol	Physical State and Its Characteristic	
N	Mechanical state $\sigma_{ij}$	$u_{n}^{eff}\overset{\sigma_{ij}\to \;\sigma_{\lim}}{\underset{}{\to }}u_{0}$
T	Thermodynamic state $T_{\Sigma }$	$u_{T}^{eff}\overset{T_{\Sigma }\to T_{S}}{\underset{}{\to }}u_{0}$
MTD	Mechanothermodynamical state $\sigma_{ijT}$, $T_{\Sigma }$	$u_{\Sigma }^{eff}\overset{\sigma_{ijT}\to \;\sigma_{\lim}(T)}{\underset{\underset{T_{\Sigma }\to T_{S}}{}}{\Rightarrow }}u_{0}$
tMTD	Mechanothermodynamical state in time $\sigma_{ijT}$, $T_{\Sigma }$, t	$u_{\sum }^{eff}\overset{\sigma_{ijT}\to \;\sigma_{\lim}(T)}{\underset{\underset{\begin{array}{c} t\to t_{\lim} \end{array}}{}}{⇛}}u_{0}$

**Table 3:** Specification of the characteristics and their physical signs of the limiting state.

Criterion	Condition of Reaching the Limiting State	Physical Sign
L1	σ_lim_ = σ*_u_* σ*_u_–*stress limit at tension	Static fracture
L2	σ_lim_ = σ_–1_ σ_–1_–mechanical fatigue limit	Fatigue fracture (into parts)
L3	σ_lim_= *p_f_* *p_f_ –*limiting contact pressure at rolling	Pittings of critical density (critical depth), excessive wear
L4	σ_lim_ = τ*_f_* τ*_f_–*limiting stress at sliding	Limiting wear
L5	$\sigma_{\lim}=\left\{ \begin{array}{l} \sigma_{-1p}\\ \sigma_{-1\tau } \end{array}\right.$ σ_–1*p*_ σ_–1τ_–limiting stress during the direct effect implementation [2]	Fatigue fracture (into parts) depending on the contact pressure (subscript *p*) at rolling or depending on the friction stress (subscript τ) at sliding (direct effect in tribo-fatigue)
L6	$\sigma_{\lim}=\left\{ \begin{array}{l} p_{f\sigma }\\ \tau_{f\sigma } \end{array}\right.$ *p_f_*_σ_, *τ_f_*_σ_–limiting stresses during the inverse effect implementation [2]	Pittings of critical density (critical depth) or excessive wear at rolling or sliding depending on the level of cyclic stresses σ (subscript σ) (inverse effect in tribo-fatique)
L7	σ_lim_ = σ_–1*q*_ σ_–1*q*_–fretting fatigue limit	Fatigue fracture at fretting corrosion and (or) fretting wear
L8	σ_lim*T*_ = σ_–1*T*_ σ_–1*T*__isothermal fatigue limit	Limiting state depending on temperature (isothermal fatigue)
L9	*T*_lim_ = *T_s_* *T_s__*melting point	Thermal (thermodynamic) damage
L10	*t*_lim_ = *t_c_* *t_c__*longevity	Time (physical) prior to the onset of the limiting state on the basis of any sign

**Table 4:** Analysis of the main characteristic of polymer materials on the basis of the experimental data.

Material and Reference	$\begin{array}{c} u_{0},\\ \frac{\mathrm{kJ}}{\mathrm{mol}} \end{array}$	$\begin{array}{c} \frac{a_{T}}{a_{n}},\;\frac{{\mathrm{MPa}}^{2}}{K}\\ \left(\frac{\mathrm{kJ}}{\mathrm{mol}\cdot K}/\frac{\mathrm{kJ}}{\mathrm{mol}\cdot {\mathrm{MPa}}^{2}}\right) \end{array}$	Tests Data	
			$\frac{K}{\sigma_{b},\;\mathrm{MPa}}$	Sample Size
Polyethylene high-density film (HDPF), grade 20806-024	108	$\frac{0.275}{2.94\cdot 10^{-4}}$	$\frac{275\ldots 383}{32\ldots 386}$	5
Polypropylene film (PF) grade 03Π10/005	119	$\frac{0.234}{1.70\cdot 10^{-4}}$	$\frac{273\ldots 423}{150\ldots 570}$	5
Hardened staple fiber, polyvinyl alcohol (PVA) “Vinol MF”	111	$\frac{0.227}{7.62\cdot 10^{-5}}$	$\frac{273\ldots 453}{80\ldots 802}$	5
Thread based on perchlorvinyl resin (PCV) grade “Chlorine”	114	$\frac{0.285}{2.56\cdot 10^{-4}}$	$\frac{273\ldots 383}{60\ldots 376}$	5
Caprone thread (PCA) (GOST 7054067)	169	$\frac{0.282}{1.68\cdot 10^{-4}}$	$\frac{275\ldots 453}{300\ldots 740}$	5
Polyethylene terephthalate film (PET) (TU 6-05-1597-72)	222	$\frac{0.342}{9.82\cdot 10^{-4}}$	$\frac{279\ldots 498}{200\ldots 362}$	4
Polyamide film PM-1 (TU 6-05-1597-72)	202	$\frac{0.297}{2.1\cdot 10^{-3}}$	$\frac{273\ldots 673}{12\ldots 240}$	7
Polystyrol (PS) at bending	281	$\frac{0.627}{2\cdot 10^{-2}}$	$\frac{77\ldots 290}{56\ldots 108}$	10
Polymetalmethacrylate (PMMA) at bending	277	$\frac{0.558}{1.74\cdot 10^{-2}}$	$\frac{77\ldots 290}{66\ldots 116}$	10
High-impact polystyrene (HIPS) at tension and torsion	277 252	$\frac{0.699}{2.53\cdot 10^{-2}}$ $\frac{0\ldots 636}{1.84\cdot 10^{-2}}$	$\frac{77\ldots 290}{48\ldots 94}$ $\frac{77\ldots 290}{50\ldots 105}$	10 10
